# Assessment of endocytic traffic and Ocrl function in the developing zebrafish neuroepithelium

**DOI:** 10.1242/jcs.260339

**Published:** 2022-09-20

**Authors:** Daniel M. Williams, Lale Gungordu, Anthony Jackson-Crawford, Martin Lowe

**Affiliations:** School of Biological Sciences, Faculty of Biology, Medicine and Health, University of Manchester, The Michael Smith Building, Oxford Road, Manchester M13 9PT, UK

**Keywords:** Endocytosis, Neuroepithelium, Ocrl, Lowe syndrome, Lrp2, Zebrafish

## Abstract

Endocytosis allows cells to internalise a wide range of molecules from their environment and to maintain their plasma membrane composition. It is vital during development and for maintenance of tissue homeostasis. The ability to visualise endocytosis *in vivo* requires suitable assays to monitor the process. Here, we describe imaging-based assays to visualise endocytosis in the neuroepithelium of living zebrafish embryos. Injection of fluorescent tracers into the brain ventricles followed by live imaging was used to study fluid-phase or receptor-mediated endocytosis, for which we used receptor-associated protein (RAP, encoded by *Lrpap1*) as a ligand for low-density lipoprotein receptor-related protein (LRP) receptors. Using dual-colour imaging combined with expression of endocytic markers, it is possible to track the progression of endocytosed tracers and to monitor trafficking dynamics. Using these assays, we reveal a role for the Lowe syndrome protein Ocrl in endocytic trafficking within the neuroepithelium. We also found that the RAP-binding receptor Lrp2 (encoded by *lrp2a*) appears to contribute only partially to neuroepithelial RAP endocytosis. Altogether, our results provide a basis to track endocytosis within the neuroepithelium *in vivo* and support a role for Ocrl in this process.

This article has an associated First Person interview with the first author of the paper.

## Teaser

**Summary:** Live-imaging assays to track endocytosis and monitor trafficking dynamics in the zebrafish neuroepithelium show involvement of the inositol phosphatase Ocrl, a protein linked to Lowe syndrome, in this process.

## INTRODUCTION

Neuroepithelial cells and related cell types such as radial glia are polarised progenitor cells that make up the bulk of brain tissue during the early stages of brain development (Götz and Huttner, 2005; Taverna et al., 2014). These cell types respond to multiple developmental cues that guide both tissue patterning and the post-mitotic fate that neuronal progenitors commit to, in order to establish a functional organ (Götz and Huttner, 2005; Taverna et al., 2014). Multiple studies have demonstrated the importance of post-Golgi endocytic trafficking in the regulation of neuronal progenitor cell function and fate. Sensing of signals that influence neuroepithelial cell behaviour is in part mediated by receptors present at the apical surface, with cell-surface receptor levels often maintained by receptor recycling from endosomes. As an example, apical enrichment of the recycling receptor low-density lipoprotein receptor-related protein 2 (LRP2) (Nagai et al., 2003) in mammalian neuroepithelial cells shapes tissue patterning within the forebrain through endocytosis of the secreted morphogen sonic hedgehog (SHH), with the absence of LRP2 leading to an impairment of SHH signalling during early neurogenesis in mice (Christ et al., 2012; Willnow and Christ, 2017). Bound ligands internalised into endosomes from the neuroepithelial cell surface can also influence neuroepithelial cell behaviour. The asymmetric segregation of the Notch ligand DeltaD within Rab5-positive SARA endosomes has been shown to be a key neuroepithelial cell-fate determinant (Coumailleau et al., 2009; Kressmann et al., 2015; Nerli et al., 2020; Zhao et al., 2021), whereas the positioning of Rab11 recycling endosomes can control developmental signalling within the zebrafish retinal neuroepithelium and *Drosophila* sensory organ precursor cells (Clark et al., 2021; Emery and Knoblich, 2006; Furthauer and González-Gaitán, 2009). Mutations in multiple trafficking regulators that are linked to central nervous system (CNS) abnormalities in mammals underscore the importance of understanding how post-Golgi trafficking events regulate neuroepithelial cell function. Mutations in α-SNAP and ARF-GEF2 lead to an impairment of neuronal development through the overproduction or underproduction of progenitor and neuronal cells, respectively (Chae et al., 2004; Sheen et al., 2004), whereas mutations in enzymes that regulate phosphoinositide signalling on membranes, such as OCRL and INPP5E, lead to a variety of CNS-associated symptoms in humans, which include epilepsy, developmental delay and intellectual disability (Bökenkamp and Ludwig, 2016; Mehta et al., 2014; Parisi, 2009; Ramirez et al., 2012).

The direct study of endocytosis and downstream trafficking steps in neuroepithelial cells *in vivo* has been limited by the lack of established protocols that allow the application of many classical methods of studying endocytosis and endocytic trafficking used in cell culture. In zebrafish, the developing brain and its associated ventricular system are easily accessible and can be imaged by microscopy (Fame et al., 2020). In addition, transgenic zebrafish lines to examine the neuroepithelial endocytic pathway have been established and characterised, whereas additional studies have demonstrated the accessibility of the hindbrain ventricle for injection of fluorescent dyes (Clark et al., 2011; Fame et al., 2020; Lowery and Sive, 2005). Here, taking advantage of these features in zebrafish, we set out to establish a semi-quantitative endocytic uptake assay to investigate endocytosis and endocytic trafficking within the developing neuroepithelium. We used a fluorescently labelled high-affinity Lrp2 ligand (RAP, encoded by *Lrpap1*) (Herz et al., 1991; Willnow et al., 1992) to assess both receptor-mediated endocytosis and downstream endocytic traffic in this tissue, and applied the assay to monitor endocytosis in *ocrl* and *lrp2* (*lrp2a*) mutant zebrafish lines. We found that RAP uptake and downstream trafficking were defective in the *ocrl* mutant neuroepithelium, with loss of Ocrl also impacting endosome size and levels of Lrp2 at the apical surface. These data suggest that the absence of Ocrl alters cell-surface levels of endocytic receptors and that disruption of receptor trafficking contributes to the neurodevelopmental defects seen in Lowe syndrome patients. Intriguingly, neuroepithelial RAP uptake still occurred in *lrp2*-null embryos, suggesting that multiple RAP-binding receptors are present at the apical surface of neuroepithelial cells. Collectively, our results illustrate that the embryonic zebrafish neuroepithelium provides an ideal system in which to visualise and study endocytic trafficking *in vivo* in the context of neuronal development.

## RESULTS

### Zebrafish neuroepithelial tissue endocytoses fluid-phase tracers injected into the hindbrain ventricle

**Fig. 1.**
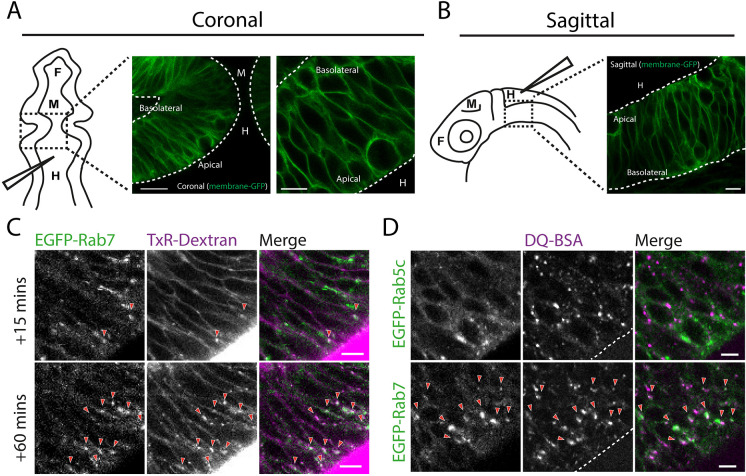
**Imaging of endocytic tracer uptake and trafficking in the zebrafish neuroepithelium.** (A,B) Schematic of tissue organisation within the developing zebrafish brain in 1 dpf embryos as viewed by confocal microscopy from coronal (A) and sagittal (B) perspectives. Dashed boxed regions indicate the regions imaged. Neuroepithelial cell membranes are labelled with transiently expressed membrane-GFP. The positions of the forebrain (‘F’), midbrain (‘M’) and hindbrain (‘H’) ventricle are indicated, with the needle showing the region injected with endocytic tracers. Dashed lines indicate apical and basolateral neuroepithelial tissue boundaries. (C) Hindbrain ventricle injection of TxR–dextran accumulates in EGFP–Rab7-labelled compartments at 15 min post injection and to a greater degree at 60 min post injection. (D) Hindbrain ventricle injection of DQ-BSA imaged in live embryos 1 h post injection shows accumulation of DQ-BSA in EGFP–Rab7-positive compartments but not in EGFP–Rab5-labelled compartments. Arrowheads point to instances of co-localisation between stably expressed EGFP–Rab5 or EGFP–Rab7 and the indicated tracer. Images are representative of a minimum of two independent experiments. Scale bars: 50 µm (A, left); 10 µm (B); 5 µm (A, right; C,D).

Zebrafish brain development begins at the early stages of embryogenesis with the rudiments of the CNS present by 10 h post fertilisation (hpf) (Kimmel et al., 1995). By 24 hpf, the overall architecture of the forebrain, midbrain and hindbrain is well defined, with tissue in these regions lining the ventricles. At this stage of development, a significant proportion of brain tissue consists of neuroepithelial cells, a key progenitor cell type from which new-born neurons and other neuroepithelial cells are derived (Götz and Huttner, 2005; Taverna et al., 2014). Viewed from both coronal (Fig. 1A) and sagittal (Fig. 1B) perspectives at 28 hpf, interkinetic nuclear migration of neuroepithelial nuclei along the apicobasal axis of the neuroepithelium gives the impression of a stratified tissue with multiple layers (Lee and Norden, 2013; Norden, 2017). Taking cells at the zebrafish midbrain–hindbrain boundary (MHB) as a representative example, however, neuroepithelial cells form a monolayer, adopting a narrow spindle-like morphology that spans the whole width of the brain, with the basal and apical processes of these cells contacting opposing surfaces. The basal process of neuroepithelial cells contacts the basal lamina adjacent to the brain wall, whereas the apical process forms junctions with neighbouring cells to integrate into an epithelial sheet that collectively makes contact with the cerebrospinal fluid of the brain ventricles (Fig. 1A,B) (Fame et al., 2020; Götz and Huttner, 2005; Norden, 2017; Taverna et al., 2014). Within these cells, the arrangement of endocytic compartments mirrors that seen in mammalian epithelial cell types. Early endosomal compartments are enriched towards the apical pole, whereas late endosomal compartments are more dispersed throughout the cell body, as previously described (Clark et al., 2011), and as visualised live in this study using EGFP-tagged Rab5c and Rab7 (encoded by *rab7a*), respectively (Fig. S1; Fig. 1D). The early and late endosomes in the zebrafish neuroepithelium are dynamic, with Rab5c-positive early endosomes tracking from the periphery towards the cell interior, albeit with some bidirectional movement observed, and Rab7-positive endosomes displaying a more striking bidirectional movement deeper within the cytoplasm (Movies 1 and 2). We also visualised the vesicle coat protein clathrin by expressing GFP-tagged clathrin light chain (CLC, encoded by *CLTA*). This revealed numerous small puncta close to or at the apical membrane and small puncta moving within the cytoplasm that likely correspond to endocytic pits and vesicles of the neuroepithelial cells (Movie 3). There were also larger dynamic puncta likely corresponding to endosomes and larger static puncta likely corresponding to the *trans*-Golgi network (Movie 3) (Liu et al., 2018). Taken together, these results indicate enrichment of early endocytic compartments at the apical region of neuroepithelial cells, as expected.

Injection of fluid-phase tracers into the zebrafish ventricular system has been used to gain insights into brain ventricle morphogenesis (Lowery and Sive, 2005; Fame et al., 2020), but so far has not been used to study endocytosis into neuroepithelial cells. We therefore set out to assess endocytosis of fluid-phase tracers. For this purpose, we injected Texas Red (TxR)-conjugated dextran into the hindbrain ventricle of zebrafish expressing the late endosome marker EGFP–Rab7 and imaged live embryos by confocal microscopy. TxR–dextran puncta were visible at 15 min post injection, likely corresponding to its presence in endosomes, which partially overlapped with Rab7 (Fig. 1C). At 1 h post injection, the TxR–dextran puncta were more numerous and brighter, and showed greater co-localisation with Rab7, consistent with continual uptake and trafficking through late endosomes (Fig. 1C). We also trialled injection of the fluid-phase tracer DQ-BSA, which becomes fluorescent upon proteolytic cleavage in late endosomes (Bright et al., 2016; Marwaha and Sharma, 2017), and performed live imaging. After 1 h of uptake, DQ-BSA was present in cytoplasmic puncta, indicating effective endocytosis and delivery to late endosomes (Movie 4). This was confirmed by co-localisation of DQ-BSA puncta with EGFP–Rab7 puncta, whereas there was little overlap between DQ-BSA and the early endosome marker EGFP–Rab5c (Fig. 1D). These results demonstrate that injected fluid-phase tracers undergo continual endocytosis from the ventricle and are transported to late endosomes and lysosomes. Thus, neuroepithelial cells can readily endocytose fluid-phase tracers injected into the hindbrain ventricle, which can be visualised by live imaging.

### A semi-quantitative assay for receptor-mediated endocytosis in zebrafish neuroepithelial tissue

**Fig. 2.**
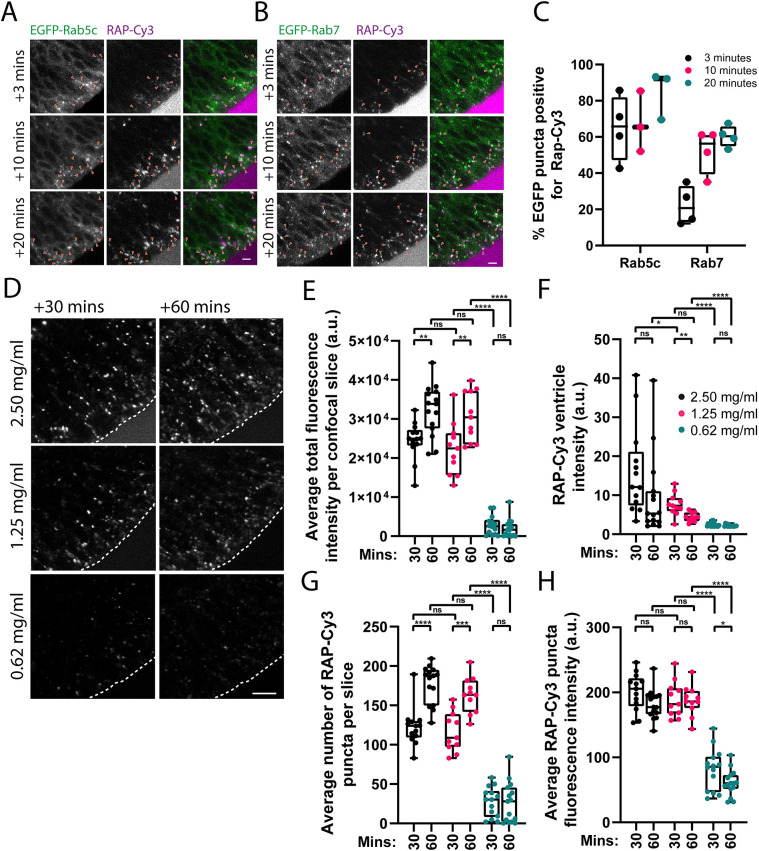
**Characterisation of neuroepithelial RAP endocytosis and endocytic trafficking.** (A,B) Confocal microscopy images of RAP–Cy3 co-localisation with stably expressed EGFP–Rab5c (A) or EGFP–Rab7 (B) in live zebrafish embryos at 3, 10 and 20 min post injection of 2.5 ng of RAP–Cy3 into the hindbrain ventricle. Arowheads indicate co-localisation between RAP–Cy3 and EGFP–Rab5c or EGFP–Rab7. Scale bars: 5 µm. (C) Quantification of co-localisation of RAP–Cy3 and EGFP–Rab5c (*n*=3), and RAP–Cy3 and EGFP–Rab7 (*n*=4) at the indicated timepoints. Each datapoint represents one individual embryo. Error bars show the s.d. (D) Representative confocal microscopy images from live imaging of RAP–Cy3 accumulation in neuroepithelial cells at 30 and 60 min post injection of 2.5, 1.25 or 0.625 mg/ml RAP–Cy3. Scale bar: 10 µm. (E–H) Quantification of the average total fluorescence intensity per confocal slice (E), RAP–Cy3 ventricle intensity at 30 or 60 min (F), the average number of RAP–Cy3 puncta per confocal slice (G) and the average segmented RAP–Cy3 punctum fluorescence intensity (H) post hindbrain injection of the indicated concentrations of RAP–Cy3 (2.5 mg/ml, *n*=15; 1.25 mg/ml, *n*=11; 0.625 mg/ml, *n*=15). a.u. arbitrary units. ns, not significant; *P<0.05; **P<0.01; ***P<0.001; ****P<0.0001. Statistical comparisons between groups were made using two-tailed unpaired Student's *t*-test.

Bulk endocytosis has been observed previously in the zebrafish neuroepithelium using the lipophilic dye FM4-64 (Clark et al., 2011), and we have also visualised fluid-phase endocytosis, as described above. However, in order to directly visualise receptor-mediated endocytosis, a specific ligand is required. We decided to use receptor-associated protein (RAP), which is a ligand for low-density lipoprotein receptor-related protein (LRP) family of receptors, of which LRP2 is known to be enriched on the apical pole of neuroepithelial cells (Kur et al., 2011; McCarthy et al., 2002; Spoelgen et al., 2005). Fluorescently conjugated RAP (RAP–Cy3) was injected into the hindbrain ventricle, and its endocytosis and trafficking in neuroepithelial cells were assessed by live imaging. RAP endocytosis into apically localised endosomes (Movies 5 and 6) could be detected as early as 3 min post injection and the amount internalised increased over time as expected (Fig. S2A,B; Movie 5). At 3 min post injection, there was significant overlap of RAP with EGFP–Rab5c and little co-localisation with EGFP–Rab7, indicating delivery to early but not late endosomes at this timepoint (Fig. 2A–C). At 10 and 20 min post injection, the co-localisation of RAP with EGFP–Rab5c remained constant, whereas it increased with EGFP–Rab7, indicating continued delivery into early endosomes and delivery of the ligand to late endosomes (Fig. 2A-C). We also observed similar co-localisation kinetics with RAP and CLC to those for Rab5c, consistent with the presence of RAP in early endocytic intermediates (Fig. S3A,B). At 1 h post injection, RAP was present in larger puncta that exhibited dynamic bidirectional movement through the neuroepithelial cell cytoplasm and occasionally in elongated tubular structures oriented along the apicobasal axis (Movie 7; Fig. S2C). There was a high degree of overlap with LAMP1–GFP, indicating that these structures corresponded to lysosomes (Movie 8). Interestingly, the lysosomes appeared to undergo fusion and tubulation/fission events, and RAP could often be seen in these intermediates (Movie 8). Blocking endocytosis with the small-molecule dynamin inhibitor Dyngo4a (McCluskey et al., 2013) greatly reduced RAP uptake into the neuroepithelium, indicating that neuroepithelial RAP endocytosis occurs by a dynamin-dependent process, likely clathrin-mediated endocytosis, as described previously for LRP2–RAP complexes (Fig. S4) (Czekay et al., 1997). Thus, as with the fluid-phase markers DQ-BSA and TxR–dextran, the receptor ligand RAP is also endocytosed by neuroepithelial cells and follows a similar itinerary, accumulating first in early endosomes before being transported to late endosomal and lysosomal compartments.

To quantify RAP endocytosis, we titrated the concentration injected and measured a number of parameters based on the amount of RAP fluorescence within the neuroepithelial tissue. Injection of 2.5 mg/ml and 1.25 mg/ml of RAP into the hindbrain ventricle yielded higher total fluorescence intensity values per area of tissue quantified at 30 and 60 min post injection, compared to injection of 0.625 mg/ml RAP (Fig. 2D,E). Due to the presence of excess RAP in the ventricle for prolonged periods of time post injection (Fig. 2F), injection of 2.5 mg/ml or 1.25 mg/ml RAP led to an increase in the amount of tissue fluorescence from 30 to 60 min (Fig. 2D,E). This increase was not seen using 0.625 mg/ml RAP (Fig. 2D,E), likely due to depletion of the limited amount of ligand in the ventricle (Fig. 2F). Similarly, increasing concentrations of RAP lead to an increase in the number of detectable puncta within neuroepithelial cells (Fig. 2G), likely as a result of RAP accumulating in endosomal and lysosomal compartments over time. However, we could not detect a statistically significant difference in either the total fluorescence intensity or number of RAP puncta following injection of 2.5 mg/ml or 1.25 mg/ml RAP. This might be because concentrations of 1.25 mg/ml and above saturate the available neuroepithelial RAP-binding receptors for prolonged periods of time post injection and lead to similar levels of RAP uptake. As an additional quantitative parameter of RAP endocytosis, we divided the total fluorescence intensity at each concentration by the total number of segmented puncta to give an average punctum intensity. As expected, the average punctum intensity was lower for 0.625 mg/ml RAP compared to that for higher concentrations (Fig. 2H). Collectively, these results demonstrate that RAP endocytosis at the neuroepithelium can be quantified and reliably detect the anticipated differences in endocytic uptake.

### *ocrl* mutant embryos display disrupted neuroepithelial RAP endocytosis and trafficking

We next applied the RAP uptake assay to an *ocrl* mutant zebrafish line to examine whether neuroepithelial RAP endocytosis and trafficking is impaired in the absence of Ocrl. Ocrl is an inositol-5-phosphatase that acts on pools of phosphatidylinositol 4,5-bisphosphate, and its activity is coupled to membrane-remodelling events at multiple stages of the endocytic pathway, which includes clathrin-mediated endocytosis (Choudhury et al., 2009; Erdmann et al., 2007; Nández et al., 2014), receptor recycling from endosomes (Choudhury et al., 2005; van Rahden et al., 2012; Vicinanza et al., 2011) and lysosome fusion (De Leo et al., 2016; Wang et al., 2021; Zhang et al., 1998). Mutations in *OCRL* are responsible for Lowe syndrome and Dent 2 disease, which is an X-linked multi-systemic disorder that predominantly affects the eyes, kidney and CNS (Bökenkamp and Ludwig, 2016). *ocrl* mutant zebrafish have previously been shown to display multiple CNS phenotypes matching those seen in Lowe syndrome, including cystic brain lesions within white-matter regions and increased sensitivity to febrile seizures during early development (Ramirez et al., 2012). These phenotypes were potentially linked to altered rates of proliferation and apoptosis within the zebrafish CNS at 1[Supplementary-material sup1]day post-fertilisation (dpf) (Ramirez et al., 2012). As neuroepithelial cells are the major proliferative cell type found in the brain during the early stages of neurogenesis, we hypothesised that loss of Ocrl function might perturb endocytic trafficking within neuroepithelial cells and contribute to the aberrant CNS development seen in o*crl* mutant zebrafish.

For RAP uptake experiments, we used an *ocrl* mutant (sa11582) obtained from the zebrafish mutation project (Kettleborough et al., 2013), containing a G to T mutation in a splice acceptor site at the boundary of intron 16–17 and exon 17, which encodes part of the *ocrl* ASH domain (Fig. S5A). This mutation abolishes a SpeI restriction enzyme site present in the wild-type (WT) zebrafish *ocrl* genomic sequence, which we could use for genotyping purposes (Fig. S5B). Embryos carrying this mutation showed a complete loss of the Ocrl protein, potentially due to nonsense-mediated decay of a faulty *ocrl* transcript, and also displayed a noticeable reduction in brain size compared to that of WT controls at the same developmental stage, consistent with phenotypes seen previously in a different *ocrl* mutant zebrafish line (Ramirez et al., 2012) (Fig. S5C,D).

**Fig. 3.**
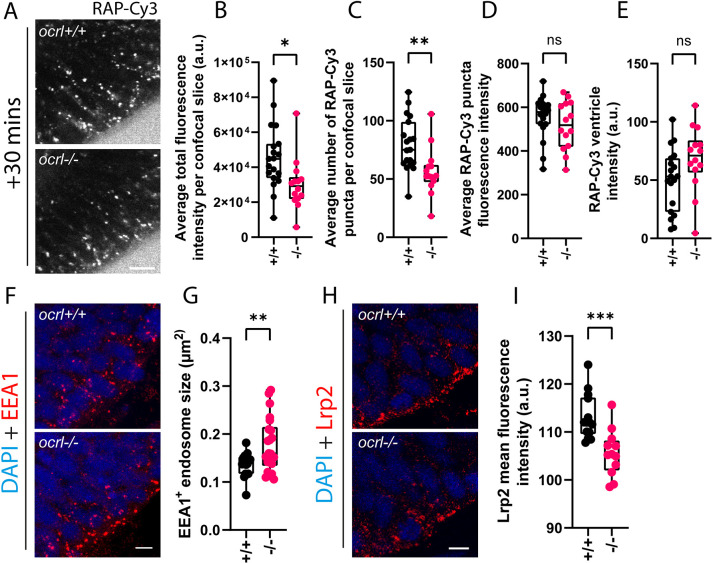
**Ocrl regulates receptor recycling and trafficking of endocytic ligands in zebrafish neuroepithelial cells.** (A) Representative confocal images of endocytic uptake of RAP–Cy3 30 min post hindbrain ventricle injection in live *ocrl* mutant embryos (*n*=14) or WT controls (*n*=19). Scale bar: 10 µm. (B–E) Quantification of the average total fluorescence intensity per confocal slice (B), average number of RAP–Cy3 puncta per confocal slice (C), average RAP–Cy3 punctum fluorescence intensity (D) and RAP–Cy3 ventricle intensity (E) at 30 min post hindbrain injection of RAP–Cy3 in WT and *ocrl* mutant embryos. (F) Representative confocal microscopy images of fixed tissue sections from WT (*n*=17) or *ocrl* mutant (*n*=22) embryos stained with antibodies against EEA1. Scale bar: 5 µm. (G) Quantification of EEA1-positive endosome size in WT and *ocrl* mutant embryos. (H) Representative confocal microscopy images of fixed tissue sections through the zebrafish hindbrain from WT (*n*=14) or *ocrl* mutant (*n*=12) embryos stained with antibodies against Lrp2. Scale bar: 5 µm. (I) Quantification of the mean Lrp2 fluorescence intensity from WT or *ocrl* mutant embryos. a.u. arbitrary units. ns, not significant; *P<0.05; **P<0.01; ***P<0.001. Statistical comparisons between groups were made using two-tailed unpaired Student's *t*-test.

Endocytosis of RAP was assessed in *ocrl* mutant embryos at 30 min following injection of 2.5 mg/ml RAP, as at this timepoint and concentration, RAP would not have been depleted from the ventricle and both early and late endosomes would be labelled. Live imaging showed that RAP uptake was reduced in *ocrl* mutants, with a lower total fluorescence and number of detectable puncta in neuroepithelial cells (Fig. 3A–C). Quantitation revealed a slight reduction in the average intensity of RAP puncta in *ocrl* mutant embryos, but this was not significant (Fig. 3D), and there was also a slight but not significant increase in the amount of RAP remaining in the ventricle at 30 min (Fig. 3E). RAP also did not appear to traffic as deep into the neuroepithelial cells in the *ocrl* mutant compared to WT controls, suggesting delayed transit along the endocytic pathway (Fig. 3A). In support of an effect on endosomal trafficking, EEA1-positive early endosomes were enlarged in *ocrl* mutant embryos, and similarly to RAP, these appeared to be more apically localised compared to those in WT embryos (Fig. 3F,G). Additionally, total Lrp2 abundance was reduced in the neuroepithelial cells of the *ocrl* mutant with a striking reduction at the apical surface, consistent with a defect in its endosomal trafficking, most likely in recycling back to the apical pole (Fig. 3H,I). A similar reduction in Lrp2 abundance was reported in *ocrl*-deficient renal proximal tubule cells and was attributed to defects in receptor recycling (Festa et al., 2019; Oltrabella et al., 2015).

### Ocrl localises to endocytic compartments in the zebrafish neuroepithelium

**Fig. 4.**
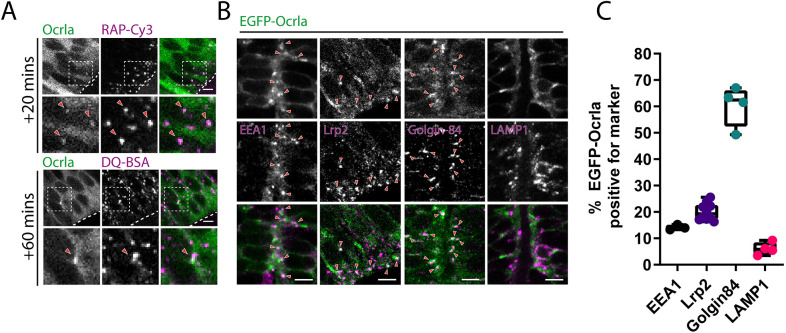
**Ocrl localises to early endocytic compartments in neuroepithelial cells.** (A) Representative confocal images of live 28 hpf zebrafish embryos transiently expressing EGFP–Ocrla after hindbrain ventricle injection of RAP–Cy3 or DQ-BSA. Regions within the dashed boxes are magnified in the lower panels. Arrowheads point to co-localisation between Ocrla signal and RAP–Cy3 or DQ-BSA. Scale bars: 5 µm. (B) Representative images of fixed tissue sections through the hindbrain of embryos transiently expressing EGFP–Ocrla and stained with antibodies against EEA1, Lrp2, Golgin-84 or LAMP1. Arrowheads indicate instances of co-localisation between Ocrla and the indicated marker. Scale bars: 5 µm. (C) Quantification of co-localisation between Ocrla puncta and EEA1 (*n*=3), Lrp2 (*n*=10), Golgin-84 (*n*=4) or LAMP1 (*n*=4) on fixed tissue sections. Each data point represents one individual embryo. Error bars represent the s.d.

We next analysed the localisation of Ocrl in the zebrafish neuroepithelium. Live imaging of embryos transiently expressing an EGFP-tagged version of the brain-specific isoform Ocrla (Ramirez et al., 2012) revealed Ocrl-containing puncta that often co-localised with endocytosed RAP and to a much lesser extent DQ-BSA, although high cytosolic levels of EGFP–Ocrla made visualisation of the puncta difficult (Fig. 4A). To further investigate whether Ocrl localises to endocytic compartments in zebrafish neuroepithelial cells, we co-labelled tissue sections through the neuroepithelium of 1 dpf embryos with various endocytic and organelle markers (Fig. 4B,C). Fixation and permeabilisation of tissue sections removed a significant fraction of the cytosolic EGFP signal, revealing multiple punctate structures throughout the cytoplasm. The majority of these structures co-localised with the Golgi marker Golgin-84, consistent with the reported localisation of Ocrl to the Golgi apparatus (Hyvola et al., 2006). A significant degree of overlap was also seen between EGFP–Ocrla and both EEA1 and Lrp2, indicating that Ocrl is recruited to early endosomal compartments within neuroepithelial cells that also likely contain Lrp2 (Fig. 4B,C). A low amount of co-localisation of EGFP–Ocrla with the late endosomal and lysosome marker LAMP1 was also observed. Taken together, these results provide evidence that Ocrl functions within the endosomal system of neuroepithelial cells to maintain normal receptor traffic.

### Neuroepithelial RAP endocytosis can occur independently of Lrp2

**Fig. 5.**
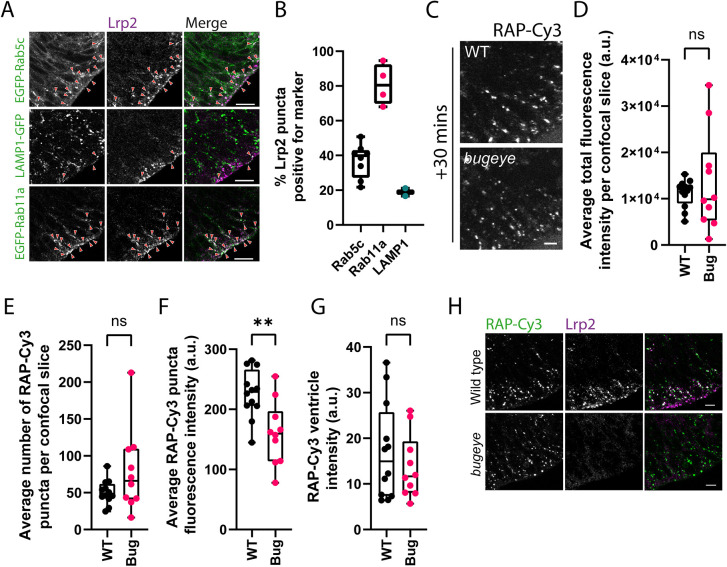
**Analysis of neuroepithelial endocytic uptake of RAP in *lrp2* knockout embryos.** (A) Tissue sections collected from embryos transiently expressing EGFP-tagged Rab5c or Rab11a, or LAMP1–GFP at 28 hpf and stained with antibodies against Lrp2 to visualise Lrp2 localisation within the zebrafish neuroepithelium. Arrowheads point to examples of co-localisation between Lrp2 and the indicated marker. Scale bars: 10 µm. (B) Quantification of Lrp2 co-localisation with EGFP-tagged Rab5c (*n*=8) or Rab11a (*n*=4) or LAMP1–GFP (*n*=2). Each datapoint represents quantification from one individual embryo. Error bars show the s.d. (C) Assessment of endocytic uptake of RAP in *lrp2* knockout *bugeye* embryos. Representative confocal microscopy images of live WT or *lrp2*-null zebrafish embryos following hindbrain ventricle injection of RAP–Cy3 imaged 30 min post injection. Scale bar: 5 µm. (D–G) Quantification of the average total fluorescence intensity per confocal slice (D), the average number of RAP–Cy3 puncta per confocal slice (E), the average RAP-Cy3 punctum fluorescence intensity (F) and RAP–Cy3 ventricle intensity (G) at 30 min post hindbrain injection of RAP–Cy3 in WT (*n*=12) and *lrp2* knockout (*n*=10) embryos. (H) Tissue sections through the zebrafish hindbrain from WT or *lrp2* knockout *bugeye* embryos injected with RAP–Cy3, fixed 30 min post injection and stained with antibodies against Lrp2. Scale bars: 10 µm. a.u. arbitrary units. ns, not significant; **P<0.01. Statistical comparisons between groups were made using two-tailed unpaired Student's *t*-test.

In the zebrafish pronephros, endocytosis of RAP is dependent upon Lrp2, which is highly abundant at the apical membrane of the proximal tubule epithelium. In this tissue, therefore, RAP can be used as a readout for Lrp2 endocytosis. However, RAP can bind to additional members of the LRP family of endocytic receptors other than LRP2 (Andersen et al., 2003; Battey et al., 1994; Kounnas et al., 1992). Thus, to determine whether RAP uptake in the zebrafish neuroepithelium is dependent on Lrp2 or other LRP family members, we first decided to localise Lrp2 in the zebrafish neuroepithelium. Co-localisation of Lrp2 was performed on cryosections of neuroepithelial tissue prepared from embryos expressing EGFP-tagged Rab5c or Rab11a, or LAMP1–GFP. In agreement with previous studies performed in mice and zebrafish (Christ et al., 2012; Kur et al., 2011), Lrp2 was abundant at the apical pole of neuroepithelial cells, localizing to puncta at the apical membrane and proximal to it (Fig. 5A). The Lrp2-containing puncta strongly co-localised with Rab11a (80%) and to a lesser extent with Rab5c (40%) (Fig. 5A,B), consistent with the transit of Lrp2 through early endosomes and its recycling via Rab11 recycling endosomes (Nagai et al., 2003; Perez Bay et al., 2016; Shah et al., 2013). A comparatively small amount of Lrp2 also co-localised with late endosomal and lysosomal compartments labelled with LAMP1–GFP, again in agreement with previous results (Christ et al., 2012).

To determine the dependence of RAP endocytosis on Lrp2 in the zebrafish neuroepithelium, RAP was injected into the hindbrain ventricle of *lrp2-*null *bugeye* embryos (Veth et al., 2011), and uptake was quantified in live embryos at 30 min post injection. Surprisingly, neuroepithelial RAP uptake was efficient in the *lrp2*-null embryos (Fig. 5C). There was no significant difference in either the total fluorescence intensity, number of RAP puncta or ventricle fluorescence intensity between WT and *lrp2*-null *bugeye* embryos (Fig. 5D,E,G). However, the average RAP punctum intensity in *lrp2*-null *bugeye* embryos was marginally lower than that in WT controls (Fig. 5F), consistent with a slight reduction in RAP endocytosis. RAP uptake was also visualised by staining of WT and *bugeye* tissue sections taken from embryos injected with RAP and fixed at 30 min post injection. This confirmed that *bugeye* embryos, despite completely lacking Lrp2 at the apical surface, endocytosed RAP into the neuroepithelial tissue at similar levels to those for WT controls (Fig. 5H). Endocytosis of RAP into the zebrafish neuroepithelium would, therefore, appear to depend on additional receptors present at the apical surface of neuroepithelial cells.

## DISCUSSION

In this study, we describe methods to assess both fluid-phase and receptor-mediated endocytosis in the developing zebrafish brain. These methods involve injection of tracer into the brain ventricle and live or fixed imaging to assess endocytosis into the surrounding neuroepithelium. Our work builds upon previous studies in zebrafish using FM4-64 to visualise bulk endocytosis (Clark et al., 2011) and a more recently published antibody-uptake approach to visualise endocytosis of the membrane-bound Notch ligand DeltaD (Zhao et al., 2021). The latter represents an assay for receptor-mediated endocytosis, albeit for an atypical membrane-associated receptor ligand. Antibody uptake assays require the availability of an antibody to the ligand or the extracellular region of the receptor of interest, and antibody binding might alter the trafficking kinetics of the proteins under investigation. In our experiments, RAP was used a more conventional soluble ligand for receptor-mediated uptake. It binds to LRP family receptors (Kounnas et al., 1992), as discussed further below, and can be easily followed by microscopy to reveal its sorting and delivery to lysosomes. Importantly, its uptake and endocytic trafficking can be quantified. Our results therefore show that quantitative assessment of endocytosis in the neuroepithelium is feasible, which provides the basis for a standardised protocol to measure this process *in vivo* under different experimental conditions.

The ability to perform live imaging of the zebrafish embryo neuroepithelium allows not only assessment of ligand uptake, but also the dynamics of trafficking within the endosomal system. Moreover, the ability to express transgenes in zebrafish embryos allows for the dual imaging of endocytic cargoes, receptors, compartment markers or trafficking machinery within the neuroepithelium (Beis and Stainier, 2006; Vacaru et al., 2014). We could exploit this property to co-visualise RAP with markers of different endosome types and lysosomes to assess traffic of the ligand through the endocytic pathway. Hence, the zebrafish embryo neuroepithelium is a highly tractable system for studying endocytic traffic *in vivo*. One limitation is that injection of tracers into the ventricle only allows for assessment of endocytosis at the apical pole; however, as for other epithelial cells, apical endocytosis appears to be a highly active process in neuroepithelial cells (Aaku-Saraste et al., 1996, 1997; Zhao et al., 2021), making it suitable for the imaging approaches described.

We applied the RAP uptake assay to study the role of Ocrl in endocytic traffic in the developing zebrafish neuroepithelium and observed several endocytic defects in *ocrl*-null embryos. There was reduced RAP uptake into the neuroepithelial cells, consistent with a role for Ocrl in clathrin-mediated endocytosis at the plasma membrane, as shown in previous *in vitro* studies (Choudhury et al., 2005, 2009; Erdmann et al., 2007; Nández et al., 2014). It also aligns with our previous work showing that clathrin-binding is important for Ocrl function in the developing brain (Ramirez et al., 2012). Our imaging experiments also indicated reduced movement of RAP from the sub-apical region to deeper within cells, suggesting reduced progression along the endocytic pathway. We also observed by confocal microscopy that early endosomes were enlarged; however, additional ultrastructural analysis of endosome size and morphology by electron microscopy would provide more definitive evidence of a swollen endosome phenotype. Collectively, these results can be explained by defective trafficking at early endosomes, as reported previously for loss of Ocrl and its interaction partners *in vitro* (Choudhury et al., 2005; Erdmann et al., 2007; Festa et al., 2019; Noakes et al., 2011; van Rahden et al., 2012; Vicinanza et al., 2011). Thus, it is likely that Ocrl operates at multiple stages of the endocytic pathway in the neuroepithelium.

Following endocytosis, Lrp2 is first trafficked to early endosomes before being sorted to recycling endosomes and returned to the cell surface for further rounds of ligand binding and uptake (Nagai et al., 2003; Perez Bay et al., 2016; Shah et al., 2013). The reduced abundance of Lrp2 at the apical pole in *ocrl* mutants would be consistent with reduced recycling from endosomes, with a similar phenomenon reported previously in renal proximal tubule cells (Festa et al., 2019; Oltrabella et al., 2022, 2015). In the absence of Ocrl, initial retention of Lrp2 in early endosomal compartments might eventually lead to its mis-sorting to lysosomal compartments, in which it can be degraded, as described previously (Oltrabella et al., 2015). This mechanism could explain the observed loss of Lrp2 signal at both the neuroepithelial cell surface and at intracellular compartments. We speculate that the neurodevelopmental defects seen in Lowe syndrome might be a result of defects in the recycling of multiple cell-surface receptors, including Lrp2, required for the sensing of signals essential for neuroepithelial cell survival or for the determination of cell fate. Further studies will be necessary to determine the precise steps and cargoes regulated by Ocrl activity within the neuroepithelial endocytic pathway.

Surprisingly, RAP endocytosis at the neuroepithelium was only marginally affected by loss of Lrp2. In zebrafish, the *lrp2* gene has been duplicated to generate *lrp2a* and *lrp2b*, but only *lrp2a* is lost in the *bugeye* mutant (Kur et al., 2011). This raises the possibility of Lrp2b being responsible for RAP uptake in *bugeye* embryos. However, previous work has shown that *lrp2b* is expressed at much lower levels than *lrp2a*, and that it does not functionally compensate for the loss of *lrp2a* during development (Kur et al., 2011). This suggests that another RAP-binding receptor is present at the apical surface of the neuroepithelium. The identity of this receptor is currently unclear. RAP can bind to other members of the LRP receptor family and several of these, such as LRP1, ApoER2 and VLDLR, are expressed in radial glia cells (Auderset et al., 2016a; Bres et al., 2020; Luque et al., 2003), a related neuroepithelial cell type that also has an apical membrane exposed to the ventricle (Götz and Huttner, 2005; Taverna et al., 2014). However, these receptors appear to be localised to the basolateral membrane of the radial glia cells (Auderset et al., 2016a,b; Boggild et al., 2018; Luque et al., 2003) and are unlikely to be the receptor for injected RAP. LRP5 and LRP6 play an important role in Wnt signalling during many developmental processes, including neural development (Angers and Moon, 2009; Gray et al., 2013; Jeong et al., 2014), and can also bind RAP, albeit with lower affinity than other members of the LRP family (Li et al., 2005). As LRP5 and LRP6 have been reported to be present at the apical surface of other epithelial cell types, they can also potentially be present at the apical membrane of neuronal precursors and, hence, might play a role in RAP uptake at the neuroepithelium (Takita and Seko, 2020; Yamamoto et al., 2017). Further studies are necessary to identify the RAP receptor at the apical membrane of the zebrafish neuroepithelium.

It is interesting to note that loss of Lrp2 does not affect neural development in zebrafish, whereas in mammals it causes forebrain malformation, owing to defects in SHH endocytosis and morphogen gradient formation (Christ et al., 2012; Kur et al., 2011). This difference might reflect different requirements for SHH signalling between mammals and teleosts, or perhaps differential requirements for LRP family receptors in endocytosis and developmental signalling between the two classes of vertebrates (Willnow and Christ, 2017). Uncovering the complement of receptors that mediate zebrafish neuroepithelial RAP endocytosis might therefore provide further insights into whether and how LRP family members contribute to neural development in zebrafish, and possibly in mammals too (Christ et al., 2012; Kur et al., 2011). Further studies using more specific ligands for the different LRP family members and specific knockouts of these family members should prove to be informative in this regard.

## MATERIALS AND METHODS

### Antibodies

The antibodies used in this study were sheep anti-zebrafish OCRL (1:200 for western blotting, custom made in the Lowe lab) (Ramirez et al., 2012), sheep anti-Golgin-84 (1:1000 for immunofluorescence or IF, custom made in the Lowe lab) (Diao et al., 2003), goat anti-EEA1 (1:400 for IF, Santa Cruz Biotechnology, SC-6415), rabbit anti-LAMP1 (1:200 for IF, Abcam, ab24170) and rabbit anti-megalin/Lrp2 (1:100 for IF). The anti-megalin/Lrp2 antibody was a kind gift from Dr Michele Marino, University of Pisa, Italy.

### Zebrafish husbandry and strains

Zebrafish were raised and maintained by the University of Manchester Biological Services Facility in accordance with the UK Animals (Scientific Procedures) Act 1986. Embryos used as WT controls were from the AB Notts strain, unless otherwise stated. Transgenic EGFP–Rab5c, EGFP–Rab7, EGFP–Rab11a and *bugeye lrp2* null mutant (*lrp2*^mw1^) zebrafish were kind gifts from Prof. Brian Link, Medical College of Wisconsin, and have been described previously (Clark et al., 2011; Veth et al., 2011). The EGFP-tagged Rab proteins were expressed from stably integrated transgenes (Clark et al., 2011). The *ocrl* mutant sa11582 was generated by N-ethyl-N-nitrosourea (ENU) mutagenesis as part of the zebrafish mutation project (Kettleborough et al., 2013). *ocrl* mutant sa11582 was backcrossed to the WT AB Notts line to generate *ocrl*^+/−^ heterozygotes, before in-crossing to obtain a pool of fish homozygous for the sa11582 *ocrl* allele. From the same in-crosses, a pool of *ocrl*^+/+^ fish was also isolated and maintained for use as sibling controls for all experiments investigating Ocrl function.

### Molecular biology and mRNA synthesis

GFP-tagged rat LAMP1, GFP-tagged human clathrin light chain A and EGFP-tagged zebrafish Rab5c, Rab7 and Rab11a were cloned into pcGlobin (kindly provided by Dr Adam Hurlstone, University of Manchester) (Ro et al., 2004) between the EcoRI and EcoRV sites using standard molecular biology techniques. Ocrla mRNA encodes zebrafish Ocrla as described previously (Ramirez et al., 2012). pCS2-GAP43-GFP was a kind gift from Prof. Nancy Papalopulu (University of Manchester) and was used to generate membrane-GFP mRNA. The mRNA for transient expression of EGFP-tagged fusion proteins was made by linearisation of plasmid DNA, followed by mRNA synthesis and purification using either T7 or SP6 mMessage transcription kits (Ambion). For transient expression, one-cell-stage embryos were injected with 1 nl of the purified mRNA solutions at the indicated concentrations: EGFP–Ocrla, 750 ng/µl; GFP–CLC, 250 ng/µl; EGFP–Rab5c, 250 ng/µl; EGFP–Rab7, 250 ng/µl; EGFP–Rab11a, 250 ng/µl; LAMP1–GFP, 250 ng/µl; and membrane-GFP, 100 ng/μl.

### Genotyping of *ocrl* mutant adult zebrafish

For genotyping of *ocrl* mutant adult zebrafish, a PCR fragment spanning the SpeI restriction site in the *ocrl* gene was amplified from genomic DNA prepared from adult fish as previously described (Wilkinson et al., 2013). The resulting 720 bp fragment was digested using SpeI (New England Biolabs) and the digested product resolved by gel electrophoresis. Fish were classified as *ocrl*^+/+^ or *ocrl*^−/−^ by the presence of a double band indicating digestion (*ocrl*^+/+^) or a single band indicating resistance to digestion (*ocrl*^−/−^).

### Western blotting

Embryos at the appropriate stage were euthanised by incubation with 0.2 mg/ml MS222 (Sigma-Aldrich) for >2 h. To remove yolk proteins, 500 μl Ginsburg fish Ringer's solution (110 mM NaCl, 3.5 mM KCl, 2.7 mM CaCl_2_·2H_2_O, 2.3 mM NaHCO_3_, 10 mM Tris-HCl pH 8.5) was added to embryos and the yolk sac disrupted mechanically through pipetting (Link et al., 2006). Following two washes in Ringer's solution, embryos were pelleted at 300 ***g*** for 1 min and the Ringer's solution removed. An appropriate volume of RIPA buffer [20 mM Tris-HCl, 150 mM NaCl, 1 mM EGTA, 1% (v/v) NP-40 substitute, 1% (w/v) sodium deoxycholate] was then added and embryos homogenised in a 1.5 ml microcentrifuge tube using a pestle. Lysates were centrifuged for 5 min at 15,000 ***g*** to pellet debris and transferred to a fresh 1.5 ml tube. Then, 6 μl of lysate was removed and protein concentration determined by bicinchoninic acid assay. Lysates were resolved by SDS-PAGE, transferred to nitrocellulose membranes, blocked in 5% (w/v) milk dissolved in PBS with 0.1% Tween (PBSTw), before being probed overnight with primary antibodies. Secondary antibody incubations were performed the next day for 1 h following three washes in PBSTw to remove unbound primary antibodies. Following secondary antibody incubation, membranes were washed another three times in PBSTw for 5 min each before imaging by chemiluminescence on a Bio-Rad Gel Doc imaging station.

### Cryosectioning and immunostaining of tissue sections

Embryos were fixed in 4% (v/v) formaldehyde in PBS (pH 7.4) at 4°C overnight or at room temperature for 2–3 h, before being rinsed three times in PBS with 0.1% (v/v) Triton X-100 (PBST) and dehydrated for a minimum of 30 min in methanol at −20°C. Embryos were then rehydrated in decreasing concentrations of 75%, 50% and 25% methanol with PBST, rinsed three times in PBST and embedded overnight in gelatin [15% (v/v) fish skin gelatin, 30% (w/v) sucrose in PBS]. Next, 12 μm sections were then cut using a Leica CM3050 cryostat, and tissue sections were collected on coated Superfrost Plus slides (Thermo Fisher Scientific). Sectioned material was dried for 1 h at room temperature before being submerged in 100% acetone at room temperature for 2 min and rehydrated in PBS for 10 min. To reduce non-specific binding of antibodies, slides were incubated with blocking solution [PBST with 10% (v/v) donkey serum] at room temperature in a humidified atmosphere. Following blocking, primary antibodies in blocking solution were added to slides and incubated for 2 h at room temperature. After primary antibody incubation, slides were rinsed in PBST six times for 5 min each. Secondary antibodies in blocking solution were then added to slides for 2 h at room temperature. Slides were again washed six times in PBST for 5 min each before mounting of coverslips using Mowiol 4-88 (Sigma-Aldrich).

### Injection and live imaging of the zebrafish neuroepithelium

To facilitate live imaging, N-phenylthiourea (Sigma-Aldrich) was added to the water at 24 h post fertilisation to inhibit melanin synthesis. For injection of endocytic tracers into the hindbrain ventricle, embryos were first anaesthetised in 0.2 mg/ml MS222, before being oriented in an agarose dish for injection and imaging as previously described (Chang and Sive, 2012). Approximately 1 nl of each tracer was injected into the hindbrain ventricle close to the midbrain–hindbrain boundary, and embryos were imaged live on either a Leica SP5 or SP8 confocal microscope with a 63× water-immersion objective. Per injection, 30 consecutive confocal planes were imaged at 1.5 µm intervals beginning at the roofplate of the dorsal MHB. The following tracers were used: TxR–dextran (1 mg/ml, Molecular Probes), DQ-BSA (2 mg/ml, Thermo Fisher Scientific) and recombinant Cy3-labelled His-tagged rat RAP (0.625–2.5 mg/ml, made in-house).

### Quantification of RAP–Cy3 endocytosis

Images were quantified using Fiji (Schindelin et al., 2012). In RAP-injected embryos, an equivalent 40 µm^2^ area of tissue within one lobe of the midbrain–hindbrain boundary was first selected. A standardised threshold was then applied to all images to exclude background fluorescence from the quantification. RAP–Cy3 puncta were segmented within each confocal plane using the ‘analyse particles’ function in ImageJ. For each confocal plane captured and analysed, the average total fluorescence intensity and number of puncta were calculated. The average total fluorescence intensity and number of puncta for each embryo injected was then calculated by taking the average total fluorescence intensity and number of puncta from each confocal plane over a minimum range of ten confocal slices starting from where the first complete cell bodies of MHB neuroepithelial cells are visible. To prevent incorporating the fluorescence remaining in the ventricle as part of the quantification, segmented puncta above a size of 5 µm were excluded from measurements used to calculate averages for each parameter. Ventricle intensity measurements were made in the first confocal plane from the dorsal MHB in which RAP fluorescence could be visualised in the ventricle.

### Statistical analysis

All statistical comparisons between groups were made in GraphPad Prism 8 or 9 using an unpaired two-tailed Student's *t*-test. Bars and error bars in data presented as a bar chart represent the mean±s.d. Values from individual embryos are overlaid. For box-and-whisker plots, boxes represent the upper and lower quartile values with median values indicated by a line inside the box. Whiskers demonstrate the highest and lowest value within each treatment group or genotype. Data presented are from a minimum of two independent experiments. *n*-values represent the total number of embryos analysed from all independent experiments.

## Supplementary Material

10.1242/joces.260339_sup1 Supplementary information
