# Supplementary material for: Assessment of endocytic traffic and Ocrl function in the developing zebrafish neuroepithelium
Source: J Cell Sci. 2022 Sep 20;135(18):jcs260339. doi: 10.1242/jcs.260339 (PMC9592051; doi:10.1242/jcs.260339)
Supplement: Supplementary information [file joces-135-260339-s1.pdf]

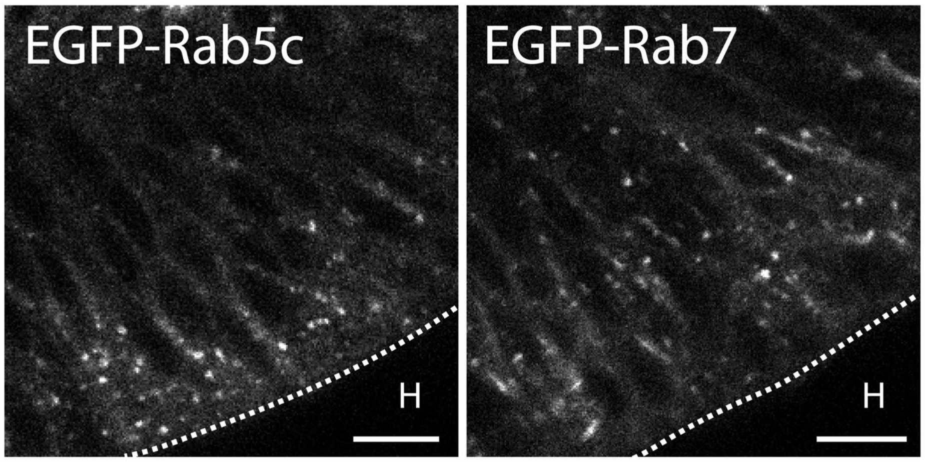

Fig. S1. Distribution of EGFP-Rab5c and Rab7 positive compartments in the zebrafish neuroepithelium. Representative confocal microscopy images showing the position of neuroepithelial early and late endosomes in 28 hpf transgenic zebrafish embryos stably expressing EGFP-Rab5c or EGFP-Rab7. Scale bar = 10 µm. Dashed line indicates boundary between apical surface of neuroepithelial tissue and the ventricle. H indicates position of hindbrain ventricle.

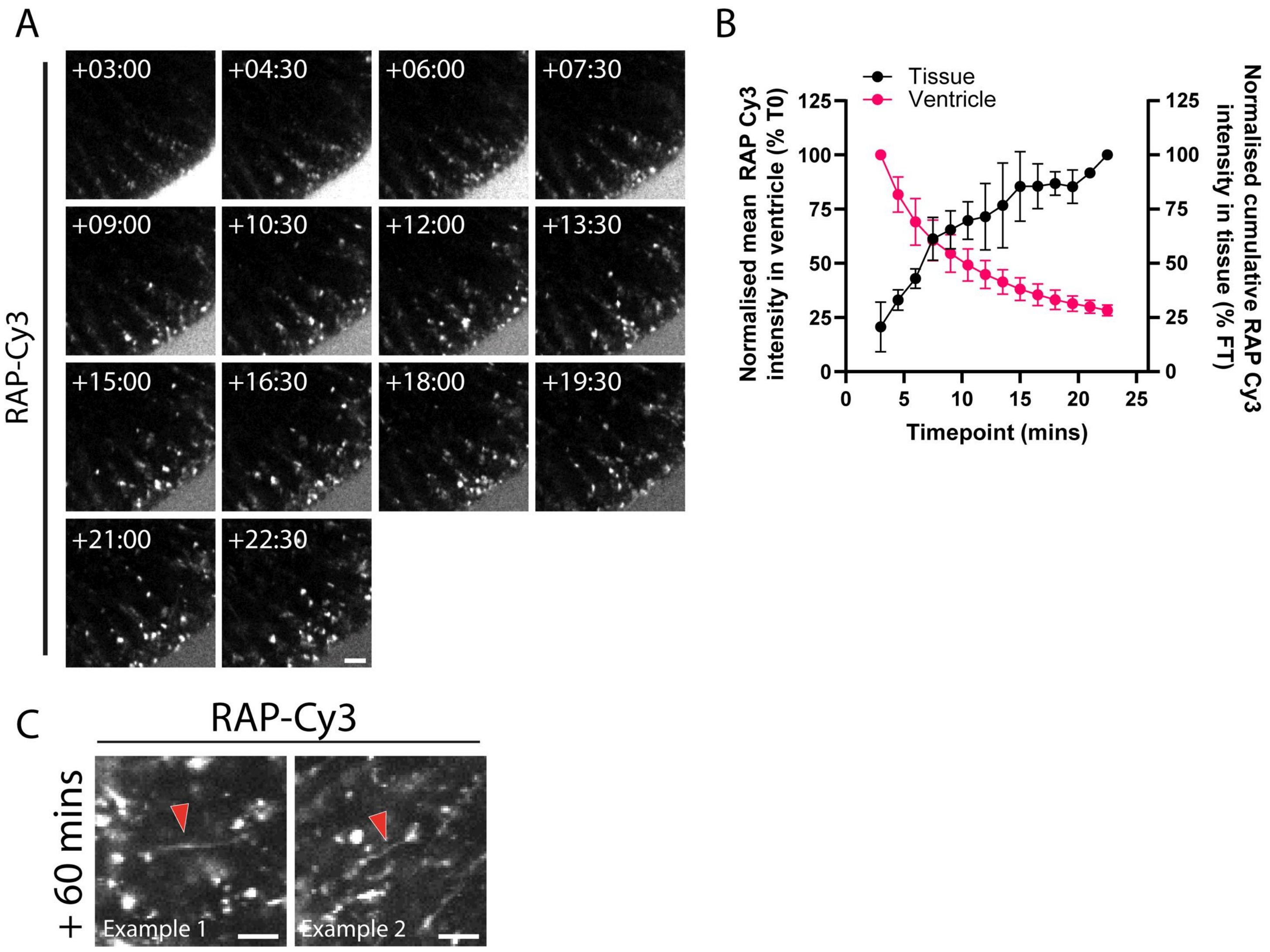

Fig. S2. Live imaging of neuroepithelial RAP-Cy3 uptake over time. (A) Representative confocal microscopy images of WT 28 hpf embryos injected with RAP-Cy3 and imaged at consecutive 1 minute 30 second intervals. Scale bar = 5 µm. **(B)** Quantification of RAP-Cy3 fluorescence intensity in the hindbrain ventricle at each timepoint post-injection and the cumulative RAP-Cy3 signal within the area of tissue pictured at each timepoint post-injection. For ventricle measurements, data were normalised to the percentage of the fluorescence intensity signal at time zero. For tissue intensity measurements of RAP, measurements were normalised to the total tissue fluorescence intensity at the final timepoint (FT) post-injection. Data presented is from 3 embryos. Error bars represent mean +/- SD. **(C)** Examples of tubular RAP-Cy3 labelled structures (indicated by arrowheads) in neuroepithelial tissue at 60 minutes post-RAP-Cy3 injection. Related to movie 7. Scale bar = 5 µm.

Journal of Cell Science • Supplementary information

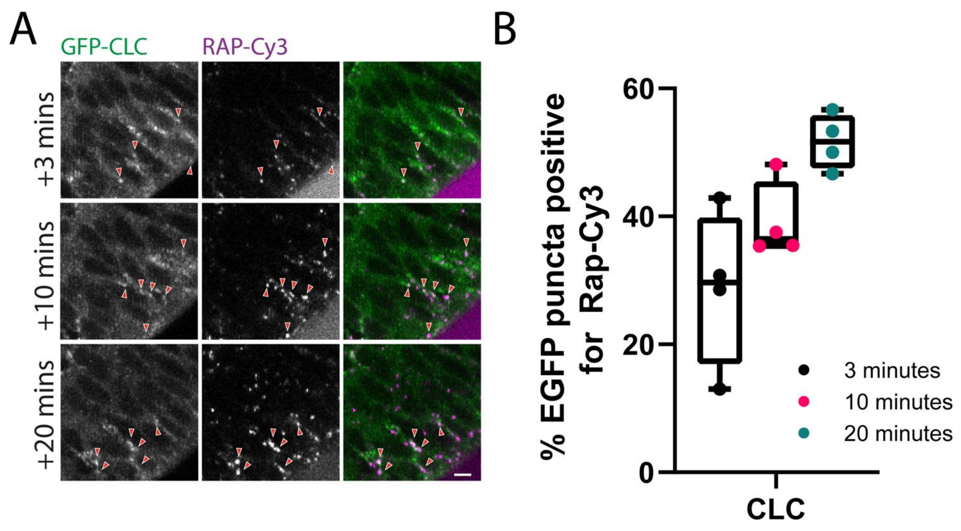

Fig. S3. Co-localisation of RAP-Cy3 with GFP-CLC in neuroepithelial cells. Confocal microscopy images of RAP-Cy3 co-localisation with **(A)** GFP-CLC in live 28 hpf zebrafish embryos at 3-, 10- and 20-minutes post-injection of 2.50 ng of RAP-Cy3 into the hindbrain ventricle. Arrowheads indicate co-localisation between RAP-Cy3 and CLC. Scale bars = 5 µm. **(B)** Quantification of co-localisation in images between RAP-Cy3 and GFP-CLC (n = 4) at the indicated timepoints. Each datapoint represents one individual embryo. Error bars = S.D.

Journal of Cell Science • Supplementary information

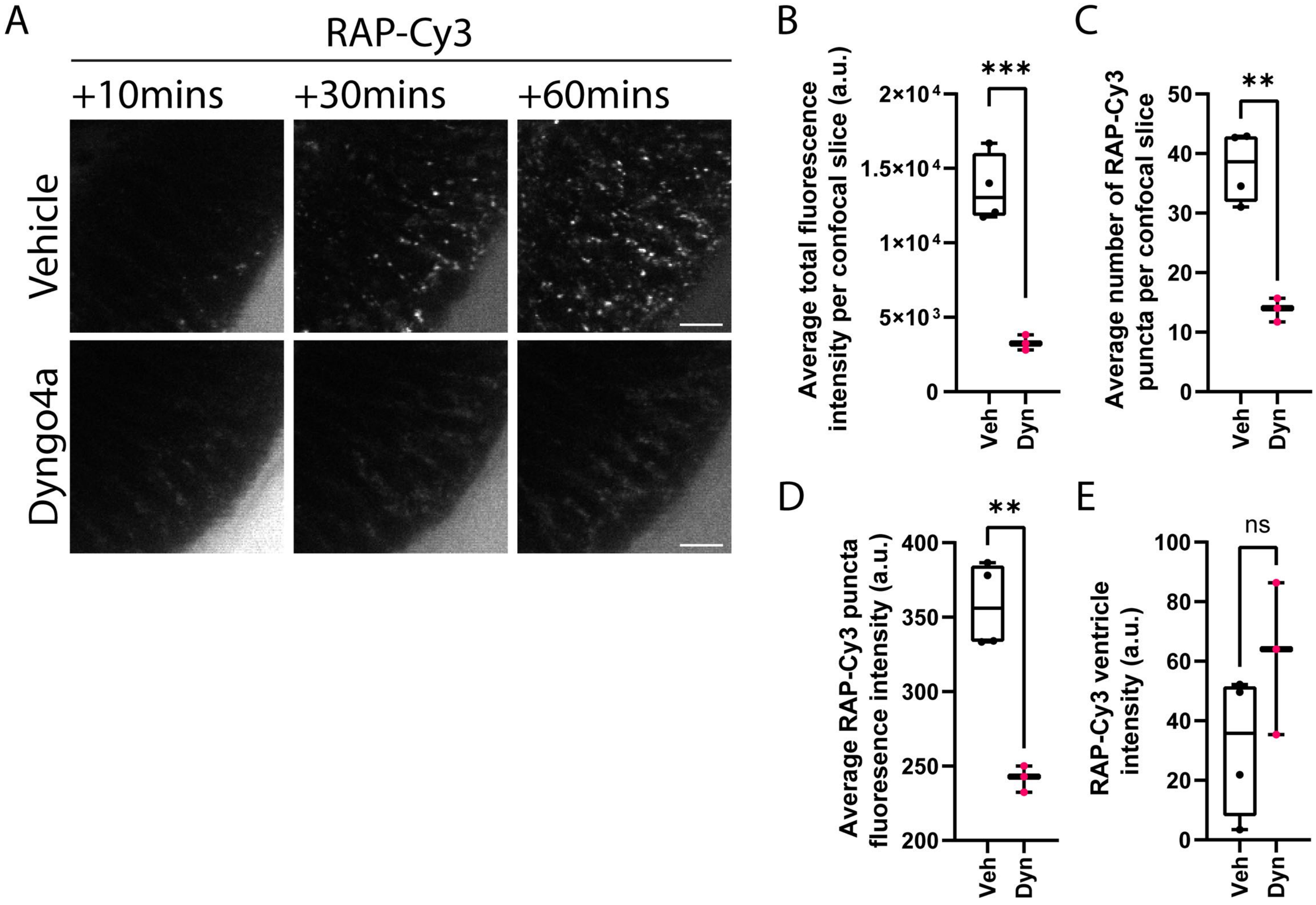

Fig. S4. Uptake of RAP into neuroepithelial tissue is mediated by a dynamin dependent endocytic process. (A) Representative confocal microscopy images of embryos pre-treated with either vehicle control (n=4) or 40 µM Dyngo4a (=3) 30 minutes prior to injection with 2.5 mg/ml of RAP-Cy3. Embryos were imaged at 10-, 30- and 60-minutes post-injection. Scale bar = 10 µm. Quantification of **(B)** the average total fluorescence intensity per confocal slice**, (C)** average number of RAP-Cy3 puncta per confocal slice**, (D)** average RAP-Cy3 puncta fluorescence intensity, and **(E)** RAP-Cy3 ventricle intensity at 30 minutes post-hindbrain injection of RAP-Cy3 in vehicle or Dyngo4a treated embryos. *<0.05; **<0.01; ***<0.001; ****<0.0001; ns, not significant. a.u. arbitrary units. Statistical comparisons between groups were made using students t-test.

Journal of Cell Science • Supplementary information

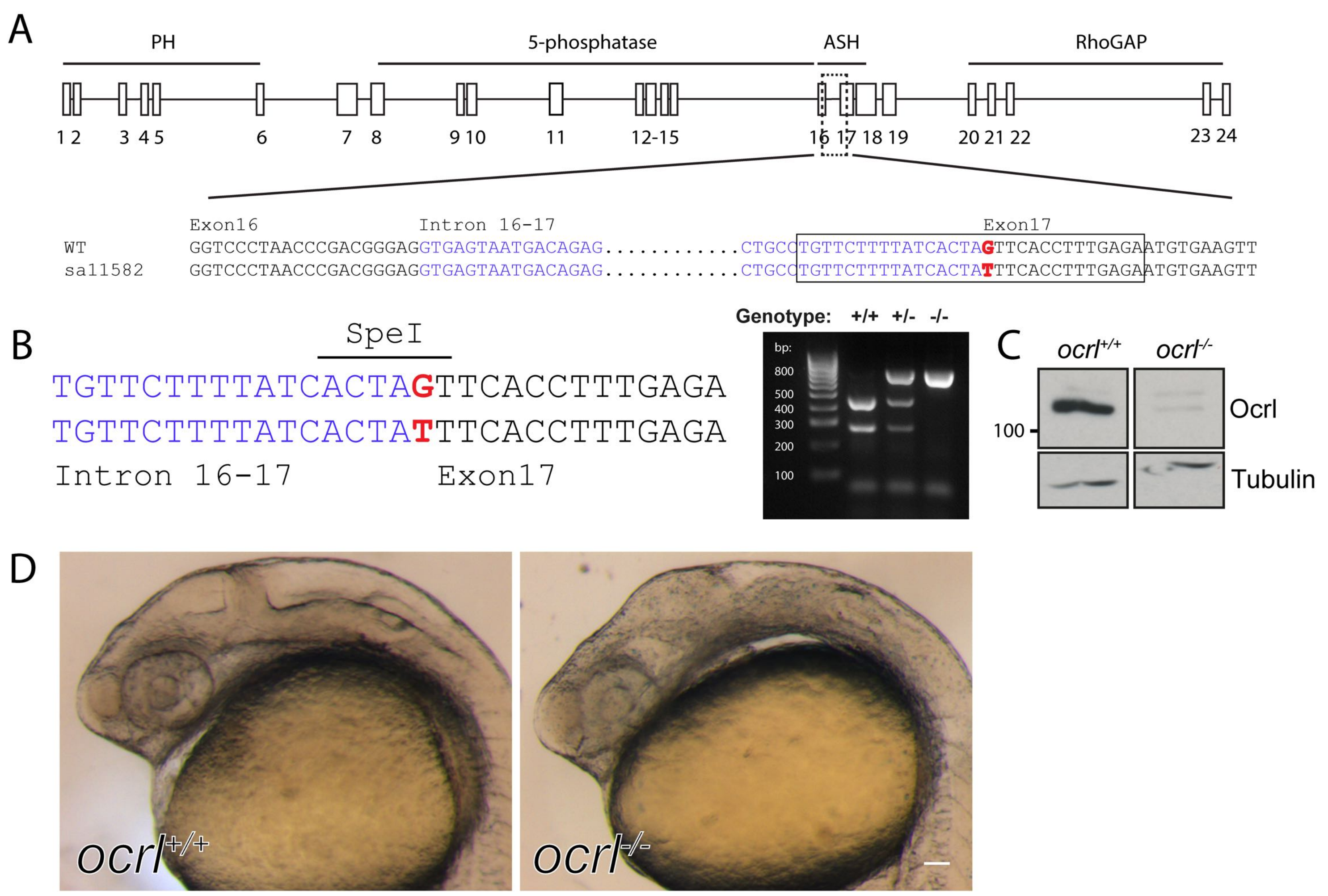

Fig. S5. Characterisation of *ocrl* mutant sa11582. (A) Overview of zebrafish *ocrl* intron and exon arrangement showing region within the *ocrl* gene affected by the sa11582 mutation. A G/T base pair mutation at the border of intron 16 and exon 17 at the start of the ASH domain disrupts a splice site between the same intron and exon. **(B)** Schematic showing DNA sequence from the boxed region in (A). G/T mutation in the ocrl mutant leads to loss of a SpeI restriction enzyme site. A PCR fragment amplified using primers flanking exon16 is resistant to SpeI digestion in *ocrl* homozygous mutant embryos. **(C)** Western blot for *Ocrl* levels in protein extracts from 28 hpf WT and *ocrl* mutant embryos. **(D)** Representative brightfield images showing brain and eye morphology in 28 hpf WT or *ocrl* sa11582 mutant embryos. ocrl mutants show a reduction in brain and eye size at the same developmental stage in comparison to WT embryos. Scale bar = 50 µm.

Journal of Cell Science • Supplementary information

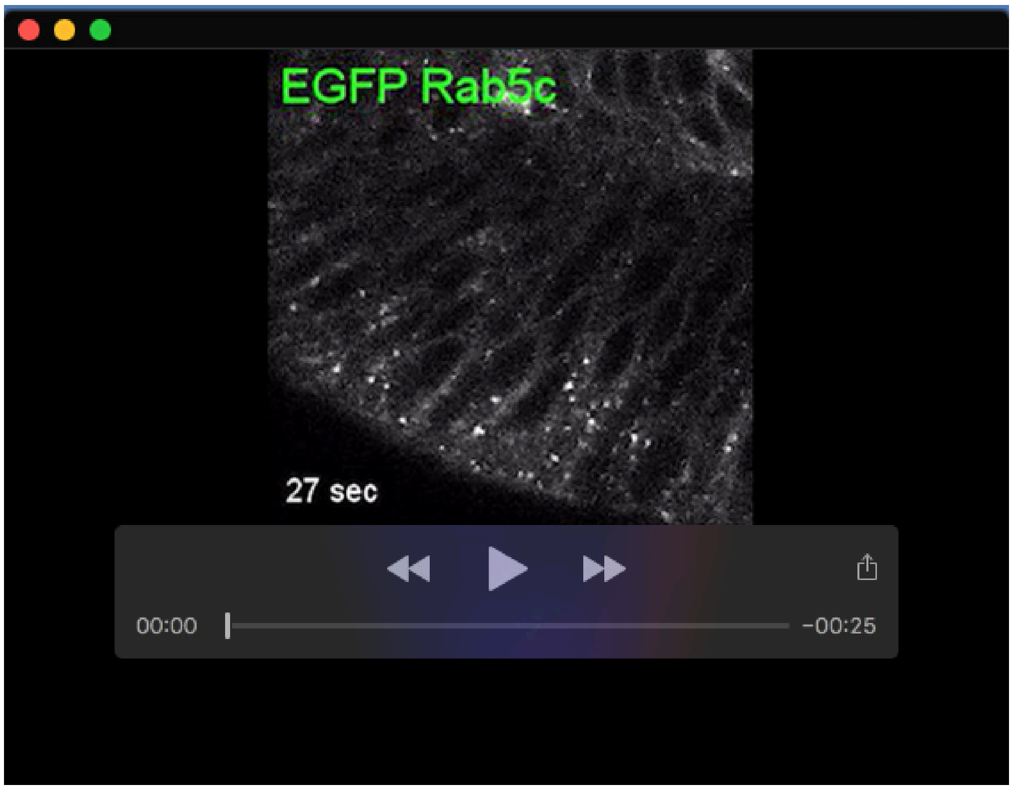

Movie 1. MHB neuroepithelial cell Rab5c dynamics. Timelapse video of 28 hpf transgenic zebrafish embryos stably expressing EGFP-Rab5c. EGFP-Rab5c puncta reside close to the apical pole and predominantly display short range movements to and from the apical membrane.

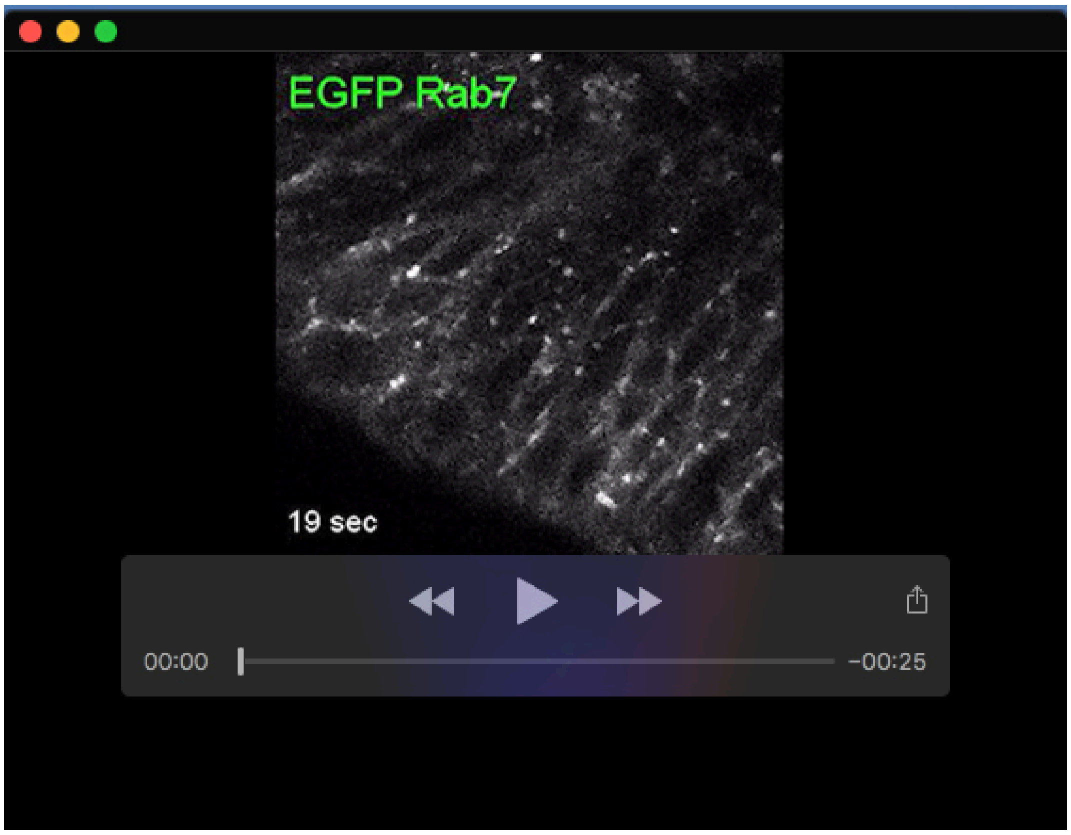

Movie 2. MHB neuroepithelial cell Rab7 dynamics. Timelapse video of 28 hpf transgenic zebrafish embryos stably expressing EGFP-Rab7. EGFP-Rab7 puncta localise deeper into neuroepithelial cells, further away from the apical surface.

Journal of Cell Science • Supplementary information

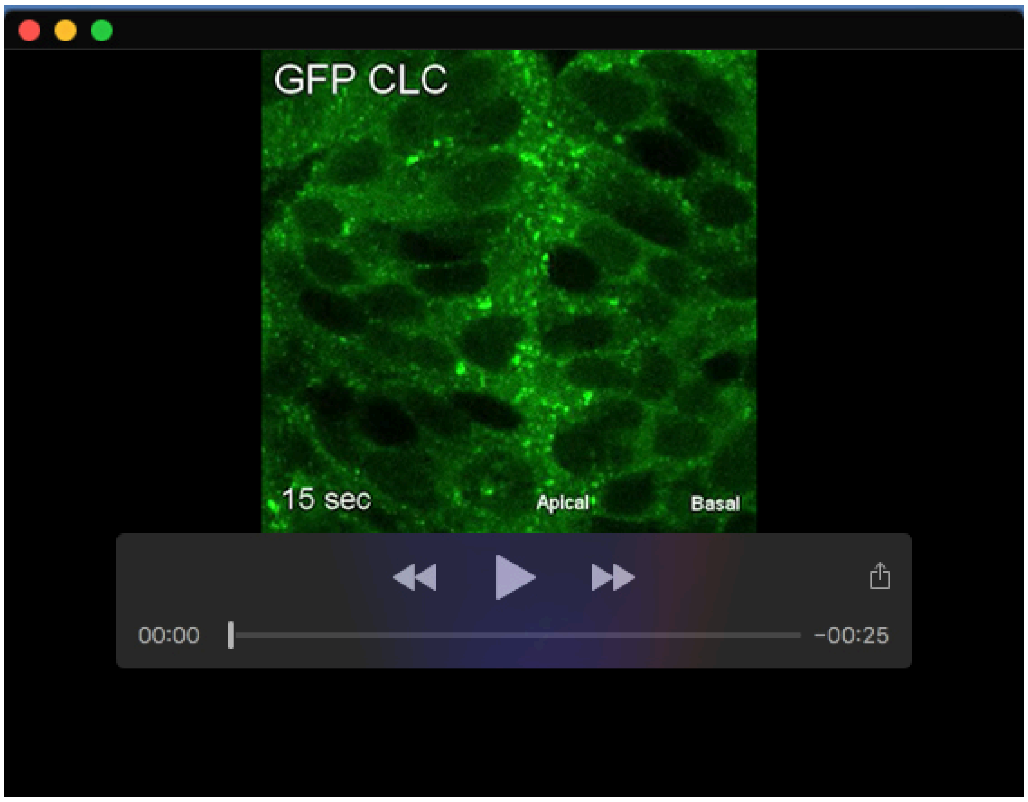

Movie 3. MHB neuroepithelial cell GFP-CLC dynamics. Timelapse video of a 28 hpf zebrafish embryo transiently expressing GFP-CLC. Embryos were imaged from a coronal perspective with the orientation of neuroepithelial cells indicated by apical and basal labels. Alongside the multiple endocytic intermediates and Golgi / endosomal pools of GFP-CLC seen in the neuroepithelial tissue, GFP-CLC can also be seen localised to a mitotic spindle in a cell undergoing division at the apical surface.

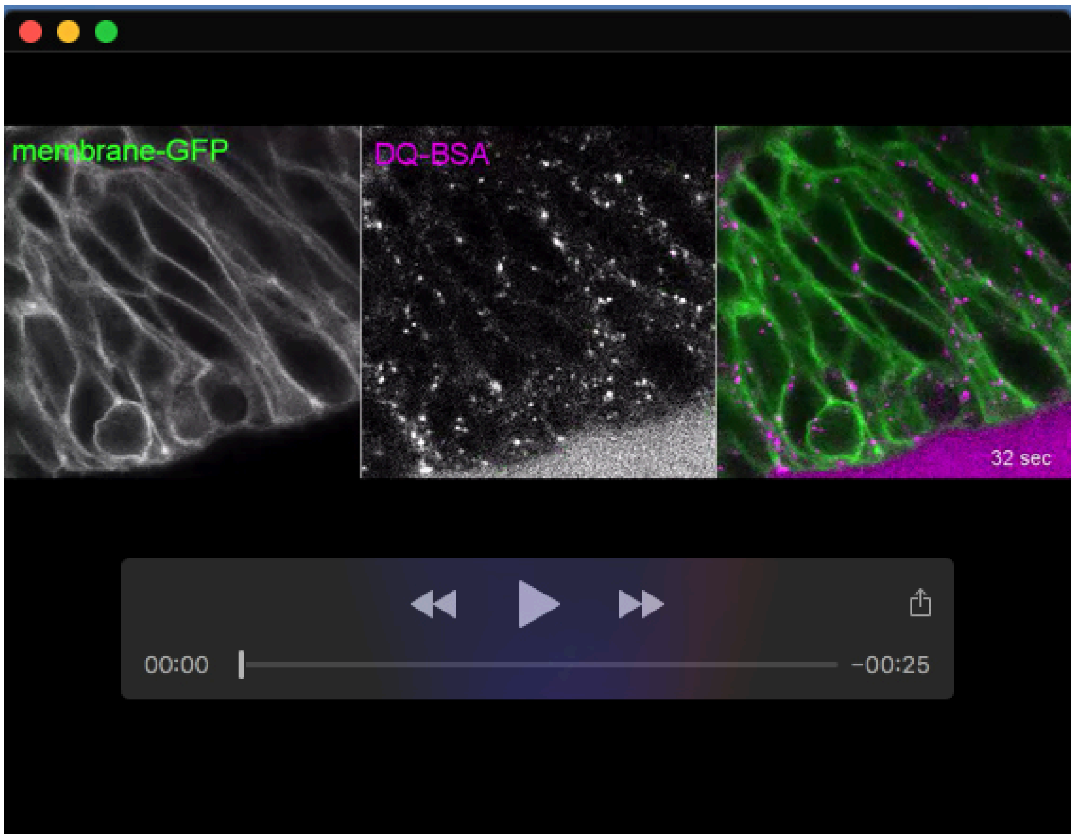

Movie 4. MHB neuroepithelial cell DQ-BSA dynamics. Timelapse video of a 28 hpf zebrafish embryo transiently expressing a membrane GFP marker injected with DQ-BSA in the hindbrain ventricle and imaged one hour post-injection. DQ-BSA puncta display dynamic long-range and short-range movements within neuroepithelial cells.

Journal of Cell Science • Supplementary information

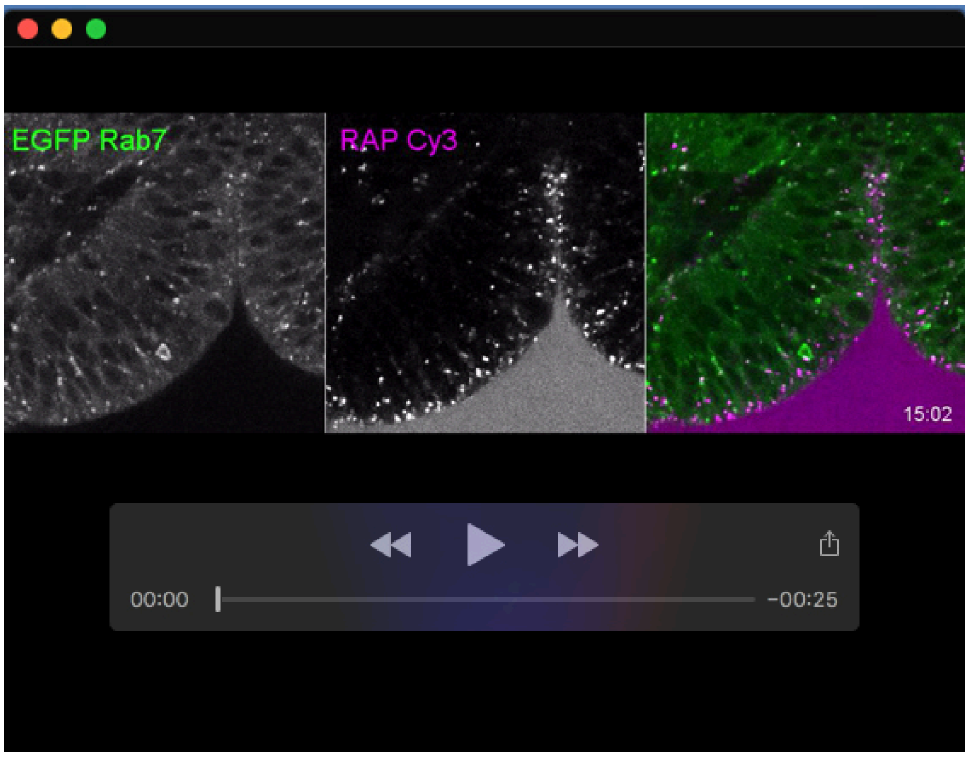

Movie 5. MHB neuroepithelial cell RAP-Cy3 uptake in transgenic EGFP-Rab7 zebrafish. Timelapse video of a 28 hpf transgenic zebrafish embryo stably expressing EGFP-Rab7, injected with RAP-Cy3. Imaging was started 3 minutes post-RAP-Cy3 injection with images acquired at 1 minute 30 second intervals. RAP intensity and the number of visible puncta within the neuroepithelium gradually increase over time whilst the amount of RAP in the ventricle is depleted.

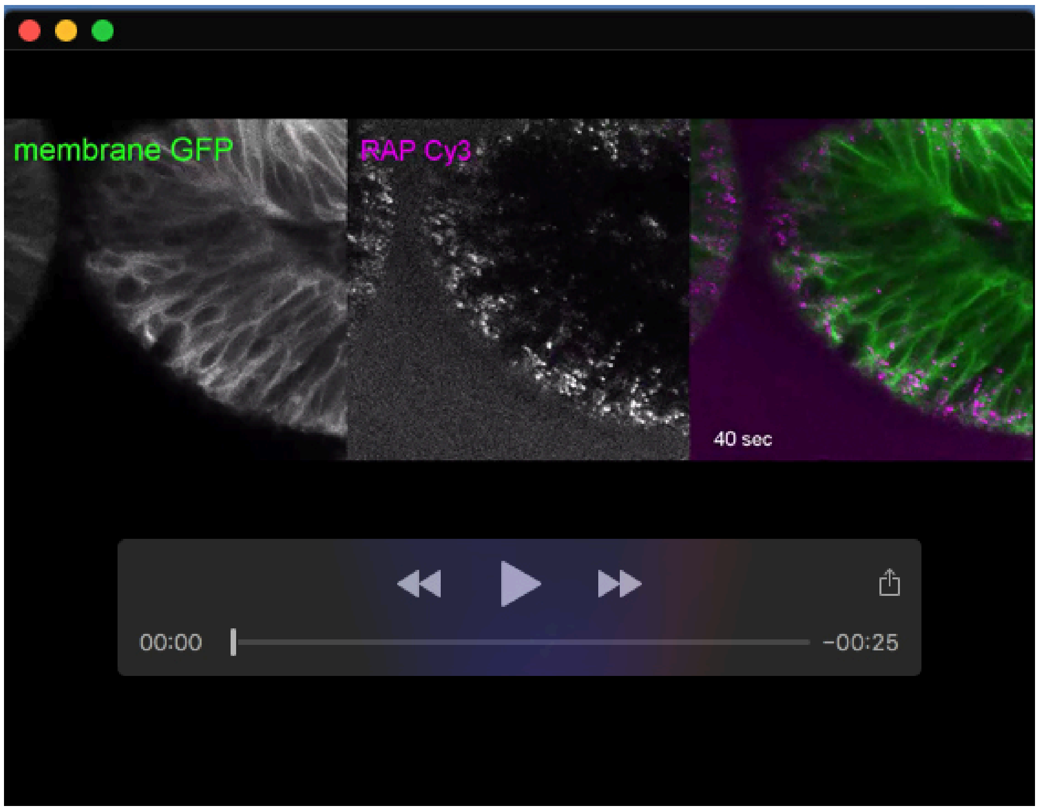

Movie 6. MHB neuroepithelial cell RAP-Cy3 dynamics at 20 minutes post-injection. Timelapse video of a 28 hpf zebrafish embryo transiently expressing membrane GFP, injected with RAP-Cy3 and imaged 20 minutes post-injection. RAP-Cy3 clusters close to the apical pole at 20 minutes post-injection, with long range movement of RAP-Cy3 towards the basolateral pole also observed.

Journal of Cell Science • Supplementary information

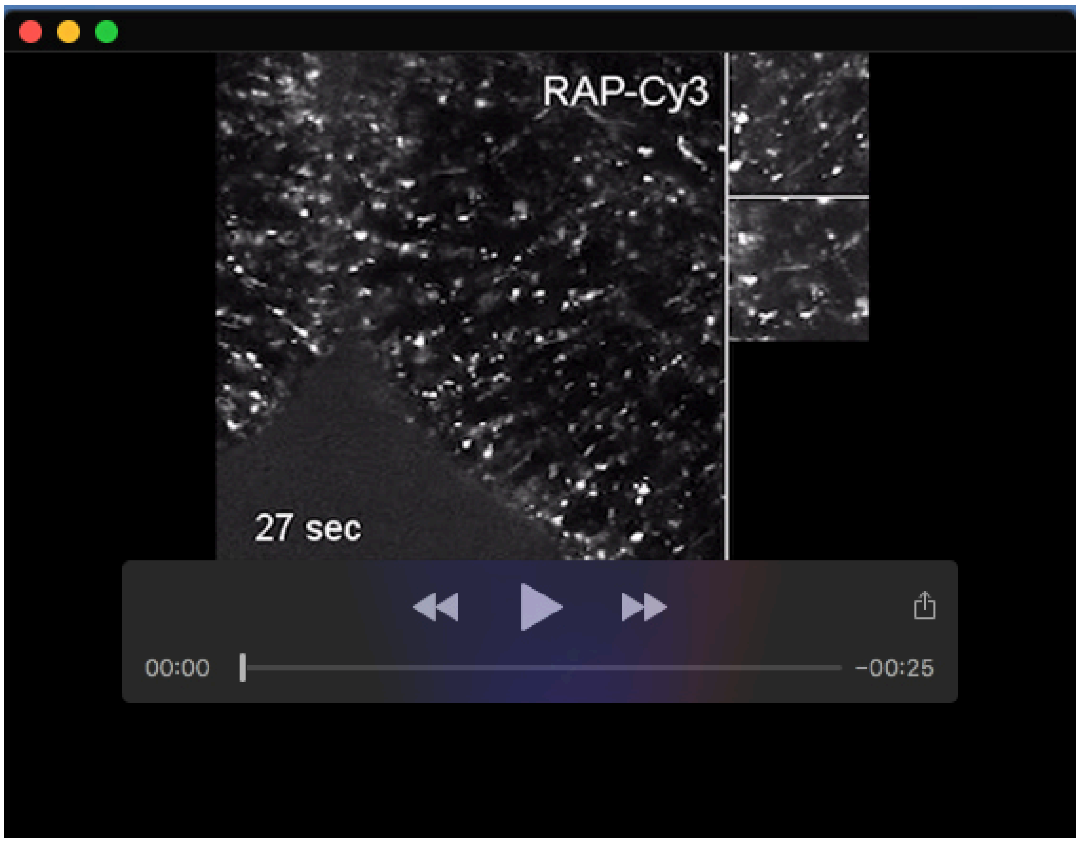

Movie 7. MHB neuroepithelial cell RAP-Cy3 dynamics at 60 minutes post-injection. Timelapse video of a 28 hpf zebrafish embryo injected with RAP-Cy3 and imaged 60 minutes post-injection. After 60 minutes, RAP is distributed equally along the apicobasal axis of neuroepithelial cells. Related to Fig. S2.

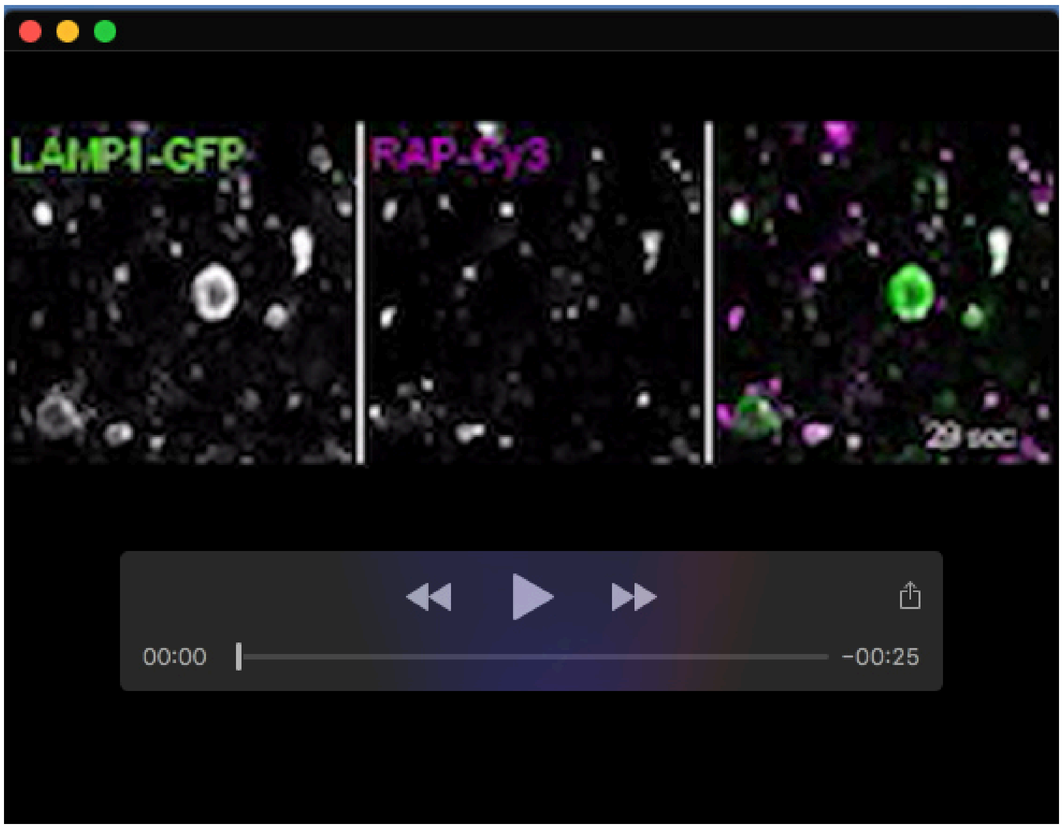

Movie 8. Sorting of RAP-Cy3 into tubules labelled with LAMP1-GFP in an MHB neuroepithelial cell. Timelapse video of a 28 hpf zebrafish embryo transiently expressing LAMP1-GFP injected with RAP-Cy3 and imaged 60 minutes post-injection (imaged sagitally). RAP-Cy3 appears in foci of larger lysosomes and is sorted into tubules emanating from LAMP1-GFP positive compartments.
