# Supplementary material for: Assessment of endocytic traffic and Ocrl function in the developing zebrafish neuroepithelium
Source: J Cell Sci. 2022 Sep 20;135(18):jcs260339. doi: 10.1242/jcs.260339 (PMC9592051; doi:10.1242/jcs.260339)
Supplement: Supplementary file 1 [file joces-135-260339_review_history.pdf]

Assessment of endocytic traffic and Ocrl function in the developing zebrafish neuroepithelium

Daniel Williams, Lale Gungordu, Anthony Jackson-Crawford and Martin Lowe
DOI: 10.1242/jcs.260339

Editor: Giampietro Schiavo

Review timeline

Original submission:	14 June 2022
Editorial decision:	7 July 2022
First revision received:	5 August 2022
Accepted:	11 August 2022

Original submission

First decision letter

MS ID#: JOCES/2022/260339

MS TITLE: Assessment of endocytic traffic and Ocrl function in the developing zebrafish neuroepithelium

AUTHORS: Daniel Williams, Lale Gungordu, Anthony Jackson-Crawford, and Martin Lowe
ARTICLE TYPE: Tools and Resources

We have now reached a decision on the above manuscript.

To see the reviewers' reports and a copy of this decision letter, please go to: https://submit-jcs.biologists.org and click on the 'Manuscripts with Decisions' queue in the Author Area. (Corresponding author only has access to reviews.)

As you will see, the reviewers gave favourable reports but raised some critical points that will require amendments to your manuscript. I hope that you will be able to carry these out because I would like to be able to accept your paper. Please get in contact if you need any clarification regarding the Reviewers' suggestions.

Please ensure that you clearly highlight all changes made in the revised manuscript. Please avoid using 'Tracked changes' in Word files as these are lost in PDF conversion.

I should be grateful if you would also provide a point-by-point response detailing how you have dealt with the points raised by the reviewers in the 'Response to Reviewers' box. Please attend to all of the reviewers' comments. If you do not agree with any of their criticisms or suggestions please explain clearly why this is so.

Reviewer 1

Advance summary and potential significance to field

In this work, 'Assessment of endocytic traffic and Ocrl function in the developing zebrafish neuroepithelium', Daniel M. Williams et al. want to establish a semi-quantitative method to describe and quantify endocytosis in the zebrafish neuroepithelium. Endocytosis plays a pivotal role

© 2022. Published by The Company of Biologists under the terms of the Creative Commons Attribution License (https://creativecommons.org/licenses/by/4.0/).

in regulating cell-cell signalling and the team describes the influence of endocytosis on e.g. Shh signalling in the introduction. Then, the authors focus on the mechanism of ocrl- and lrp2-regulated uptake – two surface receptors which are endocytosed upon external stimuli, i.e. by RAP. The authors find that in the orcl mutant fish line uptake and endocytic routing of RAP are compromised. Unfortunately, the lrp2 mutant fish does not show a significant difference in RAP uptake, although Lrp2 has been suggested as a cognate receptor for RAP.

Comments for the author

In my opinion, the manuscript is very interesting and established the zebrafish neuroepithelium as a model for studying endocytosis during neuronal development in a living organism. However, the authors should invest more time elucidating the requirement for additional receptors in RAP endocytosis to explain the lack of an lrp2 mutant phenotype. Furthermore, knock-down of Lrp2 causes a lack of Rab4-positive endosomes in the proximal pronephric duct epithelium and abrogates apical endocytosis. It would be therefore interesting to analyse if RAP can be visualized in Rab4-positive recycling endosomes. Finally, the authors speculate that altered uptake of RAP could lead subsequently to neurodevelopmental defects. I was wondering if the zebrafish could be used as a system to visualize these defects?
Specific comments
Line 133, the authors need to provide further evidence/controls to support their claim that these dyes/compounds are taken up by endocytosis in this system. For example, activation or inhibition of endocytosis can be altered genetically or by small chemical compounds. These data should be provided.
Line 145, The authors should avoid judgemental statements like “clearly visualized” in the text.
Line 206, Can the authors rescue the ocrl mutant phenotype, e.g. by microinjection of ocrl mRNA? This would strengthen the analysis of ocrl and its function in endocytic uptake and routing.
Fig. 3I the author should stick to one terminology: Lrp2 or Megalin.
Line 222, For all colocalization studies, the authors should determine and display Pearson Correlation Coefficient, PCC.

Minor points:
The authors should display their data set as a box plot, e.g. in Fig. 2c, Fig. 3F.

<u>Reviewer 2</u>

Advance summary and potential significance to field

In this study Williams and colleagues study fluid-phase and receptor-mediated endocytosis in cells lining the brain ventricles of the zebrafish. Once having established the methodology and the means of data quantification, they begin to define defects in these processes associated with an ocrl model of Lowe's syndrome (a multi-organic developmental disease, one affected organ being the brain). Specifically they examine the effect of LRP trafficking in an ocrl KO zebrafish line, revealing a partial effect on endocytosis. Overall, the data quality appear robust, with some beautiful in vivo imaging of early and late endosomal dynamics. The method development lay the foundation for further in vivo analysis of an understudied area namely the role of the endocytosis and the endocytic network in neuronal development. For this reason, and the new insight into the neurodevelopmental pathology of Lowe's syndrome, this study will be of interest to readers of the Journal of Cell Science.

Comments for the author

Minor comments:
It is stated that other LRP family members can also associate with RAP but with lower affinity, so does lowering of the RAP level provide a greater clarity on any effect of Lrp2 KO on endocytosis?
Are the Rab5 and Rab7 lines labelled endogenously? It is unclear from the methods.
Can the authors speculate on what they consider to be the mechanism for Lrp2 endocytosis and recycling. A non-specialistic reader would benefit from consideration of the potential mechanism(s) within the context of an overall model (to include significant for Lowe syndrome).

© 2022. Published by The Company of Biologists under the terms of the Creative Commons Attribution License (https://creativecommons.org/licenses/by/4.0/).

Reviewer 3

Advance summary and potential significance to field

Nowadays, the study of endocytosis, as well as of vesicle trafficking, highly relies on the use of animal models. This paper provides useful techniques to investigate endocytosis in neuroepithelial progenitor cells of zebrafish. Additionally, it proves that these techniques can be successfully applied to improve our knowledge of how endocytosis may affect the development of the CNS. For these reasons, the submitted paper is of primary interest for the readers of this journal.

Comments for the author

This paper provides a well-conceived and carefully conducted set of analyses of receptor-mediated endocytosis in neuroepithelial zebrafish cells with additional hints on Ocrl function in these progenitor cells. In my opinion, it fully deserves publication in this journal.

Minor comments and suggested revisions:

(i) lines 178-179: "Collectively, these results demonstrate that RAP endocytosis at the neuroepithelium can be quantified and reliably detect the anticipated differences in endocytic uptake". The term "quantified" may be considered an overstatement since it can only be applied to Fig.2F, where differences between the rates of endocytosis occurring at various concentrations of the injected marker are statistically significant at the same time point. This observation cannot be extended to the other statistic analyses in Fig.2.

Henceforth, I would rephrase the sentences from line 170 to the end of the paragraph to reflect this consideration.

(ii) lines 215-216: "In support of an effect on endosomal trafficking, EEA1-positive early endosomes were enlarged in ocrl mutant embryos...". The measurement of enlarged endosomes relies on confocal analyses.
It would be more appropriate to use EM for this purpose. However, since these data are not crucial for the conclusions of this paper, I advise mentioning that ultrastructural analyses may be more appropriate to draw final conclusions on this topic.

(iii) Fig. 4, panel A. The staining for Ocrla is very dim and raises doubts about a possible spillover/bleed-through of the Cy3 channel into the EGFP channel. I would suggest checking for EGFP-Ocrla expression, firstly (e.g., by WB). Then, if confirmed, you could either repeat the experiment and improve the EGFP-Ocrla detection or check whether there is any bleeding from the Cy3 channel into the EGFP channel by undertaking the appropriate controls.

First revision

Author response to reviewers' comments

Reviewer 1 Advance Summary and Potential Significance to Field...

In this work, ‘Assessment of endocytic traffic and Ocrl function in the developing zebrafish neuroepithelium’, Daniel M. Williams et al. want to establish a semi-quantitative method to describe and quantify endocytosis in the zebrafish neuroepithelium. Endocytosis plays a pivotal role in regulating cell-cell signalling and the team describes the influence of endocytosis on e.g. Shh signalling in the introduction. Then, the authors focus on the mechanism of ocrl- and lrp2-regulated uptake – two surface receptors which are endocytosed upon external stimuli, i.e. by RAP. The authors find that in the orcl mutant fish line uptake and endocytic routing of RAP are compromised. Unfortunately, the lrp2 mutant fish does not show a significant difference in RAP uptake, although Lrp2 has been suggested as a cognate receptor for RAP.

© 2022. Published by The Company of Biologists under the terms of the Creative Commons Attribution License (https://creativecommons.org/licenses/by/4.0/).

Reviewer 1 Comments for the Author...

In my opinion, the manuscript is very interesting and established the zebrafish neuroepithelium as a model for studying endocytosis during neuronal development in a living organism. However, the authors should invest more time elucidating the requirement for additional receptors in RAP endocytosis to explain the lack of an lrp2 mutant phenotype.
We thank the reviewer for these comments and agree that the identity of the additional receptor(s) mediating RAP endocytosis is an intriguing question that merits further investigation. However, these studies will require a significant amount of additional work which we feel is beyond the scope of the current study and instead will likely form the basis of future investigations.

Furthermore, knock-down of Lrp2 causes a lack of Rab4-positive endosomes in the proximal pronephric duct epithelium and abrogates apical endocytosis. It would be therefore interesting to analyse if RAP can be visualized in Rab4-positive recycling endosomes.
We thank the reviewer for highlighting this interesting point and agree that it would be of interest to investigate the effects of Lrp2 knockdown on endocytic compartments within the neuroepithelium and to further characterise the itinerary of RAP with more markers of endocytic recycling pathways. Given the co-localisation of RAP with Rab5 and the known co-localisation between Rab5 and Rab4 on sorting endosomes (Sonnichsen, De Renzis et al. 2000) it is likely that RAP would show some co-localisation with Rab4 in WT embryos. The uptake of RAP into similar punctate structures in *lrp2* null embryos suggests that RAP follows a similar itinerary in the absence of *lrp2* that would also likely overlap with Rab4 to some degree. However, whilst the itinerary of RAP within the neuroepithelium in various genetic backgrounds would certainly be of interest to examine in future studies, we do not think examining RAP co-localisation with Rab4 is absolutely necessary to support the conclusions of current study, but once more thank the reviewer for this helpful suggestion.

Finally, the authors speculate that altered uptake of RAP could lead subsequently to neurodevelopmental defects. I was wondering if the zebrafish could be used as a system to visualize these defects?
We have previously characterised a separate ocrl mutant zebrafish line and demonstrated that loss of Ocrl function impacts several aspects of neuronal development (Ramirez, Pietka et al. 2012). Similar morphological abnormalities were seen in the mutant zebrafish line used in this manuscript but further characterisation of these defects and comparison with the original mutant line was not the main focus of this study and as such, we have not at present pursued this any further.

Specific comments

Line 133, the authors need to provide further evidence/controls to support their claim that these dyes/compounds are taken up by endocytosis in this system. For example, activation or inhibition of endocytosis can be altered genetically or by small chemical compounds. These data should be provided.
We have performed an experiment where embryos were treated with the small molecule dynamin inhibitor Dyngo4a (McCluskey, Daniel et al. 2013) prior to RAP-Cy3 injection. Dyngo4a treatment greatly reduced (although did not completely abolish) RAP-Cy3 uptake over 1 hour suggesting that the majority of RAP-Cy3 uptake is through a dynamin dependent endocytic process (likely clathrin-mediated endocytosis (Czekay, Orlando et al. 1997)). This data is provided in a new Fig S4.

Line 145, The authors should avoid judgemental statements like “clearly visualized” in the text.
The text has been updated to remove judgemental statements.

Line 206, Can the authors rescue the ocrl mutant phenotype, e.g. by microinjection of ocrl mRNA? This would strengthen the analysis of ocrl and its function in endocytic uptake and routing.
During the course of our study, we attempted to rescue RAP-Cy3 uptake through microinjection of *ocrl* mRNA into *ocrl* mutant embryos. However, due to technical difficulties we abandoned these experiments. We found transient expression of o*crl* to be extremely variable and struggled to obtain sufficient numbers of embryos per experiment to inject and analyse. Thus, whilst we agree that this experiment would strengthen our conclusions

© 2022. Published by The Company of Biologists under the terms of the Creative Commons Attribution License (https://creativecommons.org/licenses/by/4.0/).

regarding the function of Ocrl in endocytic trafficking within the neuroepithelium, we unfortunately cannot provide a satisfactory dataset using this method. For future work we are considering generating a stable transgenic zebrafish line with EGFP- Ocrl under the control of a neuroepithelial specific promoter to express in an *ocrl* KO background but at present have not generated this line.

Fig. 3I the author should stick to one terminology: Lrp2 or Megalin.
We have used Lrp2 throughout the paper but had overlooked the mislabelling of Lrp2 as megalin in Fig3I. This has now been corrected.

Line 222, For all colocalization studies, the authors should determine and display Pearson Correlation Coefficient, PCC.
We have analysed co-localisation by Pearson correlation co-efficient (PCC) for all relevant images as suggested (Reviewer Figure 1). PCC analysis gives similar trends as the manual object-based quantification for RAP colocalization with Rab5c and Rab7 and for Lrp2 co- localisation with endosomal markers. However, PCC analysis gave results that do not reflect the localisation of Ocrl to the Golgi or early endosomes, producing average correlation coefficients of 0.49 and 0.43 respectively. These values are similar to the value of 0.37 for Ocrl analysed with LAMP1 where little to no overlap in signal is visible from the images themselves. Based on these results, we would prefer for all relevant figures to include our original analysis in the manuscript, whereby manually identified puncta are scored for co-localisation with the corresponding marker. This method of quantification matches the PCC analysis for Lrp2 and RAP and more accurately reflects the co-localisation of Ocrla with Golgin84 and EEA1 seen in the confocal images. This is in-line with published articles on the relative merits of pixel-based quantitation such as PCC versus object-based analysis (see for example Lagache, Sauvonnet et al. 2015).

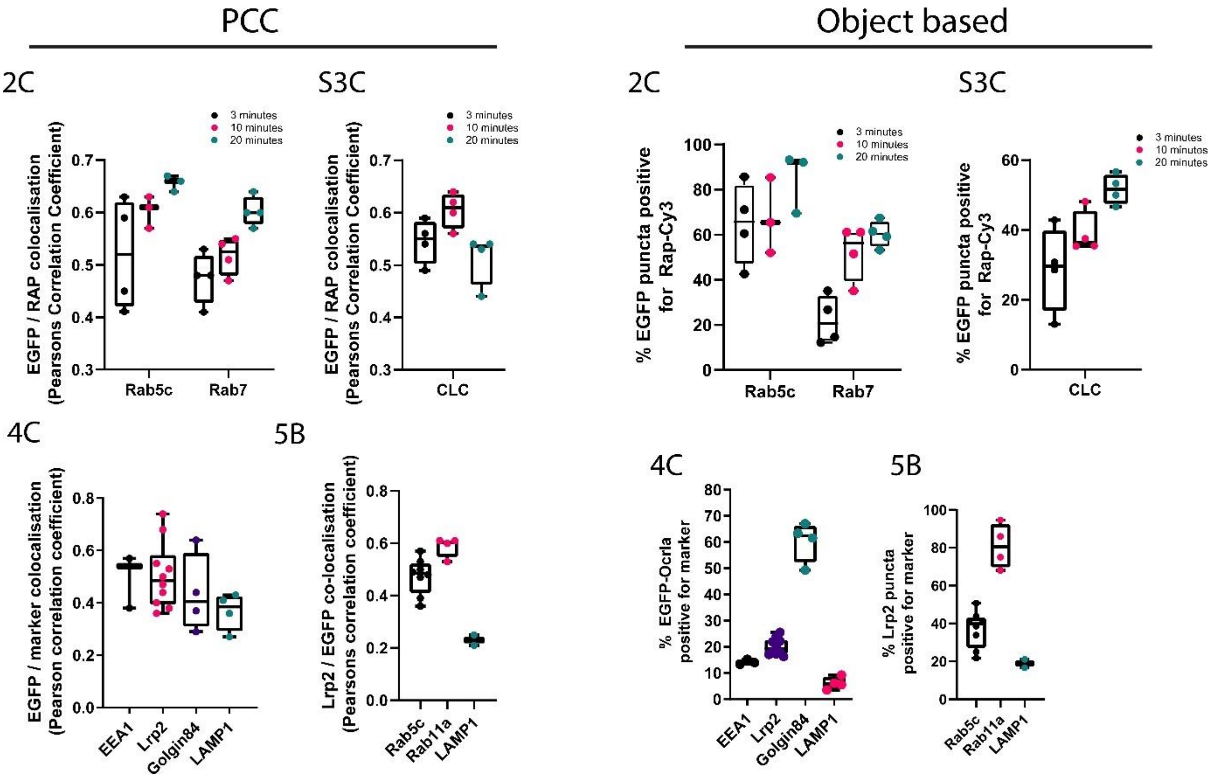

Reviewer Figure 1. Comparison of Pearson correlation coefficient analysis versus object-based colocalisation for the various markers in the indicated figures, as used in the manuscript.

Minor points:
The authors should display their data set as a box plot, e.g. in Fig. 2c, Fig. 3F.
We have changed all remaining bar charts to box plots.

© 2022. Published by The Company of Biologists under the terms of the Creative Commons Attribution License (https://creativecommons.org/licenses/by/4.0/).

Reviewer 2 Advance Summary and Potential Significance to Field...

In this study Williams and colleagues study fluid-phase and receptor-mediated endocytosis in cells lining the brain ventricles of the zebrafish. Once having established the methodology and the means of data quantification, they begin to define defects in these processes associated with an ocrl model of Lowe's syndrome (a multi-organic developmental disease, one affected organ being the brain). Specifically, they examine the effect of LRP trafficking in an ocrl KO zebrafish line, revealing a partial effect on endocytosis. Overall, the data quality appear robust, with some beautiful in vivo imaging of early and late endosomal dynamics. The method development lay the foundation for further in vivo analysis of an understudied area, namely the role of the endocytosis and the endocytic network in neuronal development. For this reason, and the new insight into the neurodevelopmental pathology of Lowe's syndrome, this study will be of interest to readers of the Journal of Cell Science.
We thank the reviewer for their positive comments on our work.

Reviewer 2 Comments for the Author...

Minor comments:
It is stated that other LRP family members can also associate with RAP but with lower affinity, so does lowering of the RAP level provide a greater clarity on any effect of Lrp2 KO on endocytosis?
This is an interesting point which we thank the reviewer for highlighting and have investigated further through additional analysis of our existing dataset for RAP uptake in *lrp2* knockout embryos. As there are small variations in the volume of RAP-Cy3 injected per embryo at a set concentration, we plotted the amount of RAP in the ventricle shortly after injection against the average puncta intensity at 30 minutes to see if lower initial RAP ventricle intensity correlates with lower RAP intensity in the tissue at later timepoints in *lrp2* null embryos versus WT. Reviewer Fig 2 shows that initial ventricle intensities of 20-30 or lower frequently lead to lower average RAP puncta intensities in *lrp2* null embryos compared to WT controls. This would suggest that at lower initial concentrations of RAP, *lrp2* knockout embryos fail to endocytose RAP as effectively as WT controls. This would be consistent with Lrp2, when present, mediating the bulk of RAP endocytosis and receptors with lower affinity for RAP mediating endocytosis in the absence of Lrp2.

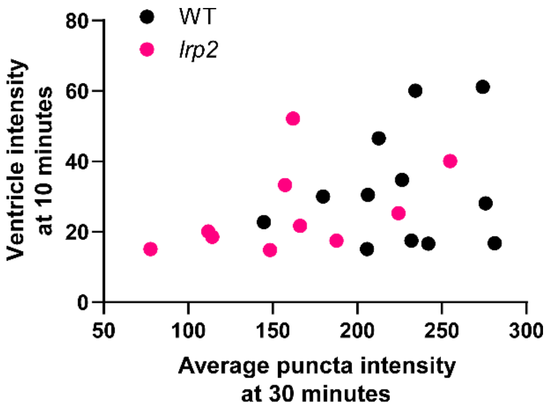

Reviewer Figure 2. Lower RAP-Cy3 ventricle intensities at early timepoints post injection lead to lower levels of RAP-Cy3 tissue accumulation 30 minutes post injection in *lrp2* null embryos versus WT controls.

Ventricle injection of LRP5 and LRP6 specific ligands/chaperones such as mesd (Hsieh, Lee et al. 2003), or knockdown or knockout of other LRP receptors expressed in the neuroepithelium may also be interesting avenues to further unravel the contributions of LRP receptors in neuroepithelial RAP endocytosis. However, these experiments would require a large amount of additional work which we feel are beyond the aims of the current study.

Are the Rab5 and Rab7 lines labelled endogenously? It is unclear from the methods.
We apologise for any confusion caused by the lack of a clear description of these zebrafish lines.

© 2022. Published by The Company of Biologists under the terms of the Creative Commons Attribution License (https://creativecommons.org/licenses/by/4.0/).

The EGFP-tagged Rab5 and Rab7 lines are expressed from transgenes as described previously (Clark, Winter et al. 2011) and are not labelled endogenously. We have updated the text in the Methods section to make this clearer to readers.

Can the authors speculate on what they consider to be the mechanism for Lrp2 endocytosis and recycling. A non-specialistic reader would benefit from consideration of the potential mechanism(s) within the context of an overall model (to include significant for Lowe syndrome).
The discussion (page 12) has now been updated to include a discussion of the mechanism of Lrp2 endocytosis and recycling and the implications for Lowe syndrome pathology in the brain.

“Following endocytosis, Lrp2 is first trafficked to early endosomes before being sorted to recycling endosomes and returned to the cell surface for further rounds of uptake (Nagai, Meerloo et al. 2003, Shah, Baterina et al. 2013, Perez Bay, Schreiner et al. 2016). The reduced abundance of Lrp2 at the apical pole in *ocrl* mutants would be consistent with reduced recycling from endosomes, with a similar phenomenon reported previously in renal proximal tubule cells (Oltrabella, Pietka et al. 2015, Festa, Berquez et al. 2019, Oltrabella, Jackson-Crawford et al. 2021). Initial retention of Lrp2 in early endosomal compartments may eventually lead to its mis-sorting to lysosomal compartments where, as described previously (Vicinanza, Di Campli et al. 2011, Oltrabella, Pietka et al. 2015), it can be degraded. This mechanism could explain the observed loss of Lrp2 signal at both the neuroepithelial cell surface and at intracellular compartments. We speculate that the neurodevelopmental defects seen in Lowe syndrome may be a result of defects in the recycling of multiple cell surface receptors, including Lrp2, required for the sensing of signals essential for neuroepithelial cell survival or the determination of cell fate.”

Reviewer 3 Advance Summary and Potential Significance to Field...

Nowadays, the study of endocytosis, as well as of vesicle trafficking, highly relies on the use of animal models. This paper provides useful techniques to investigate endocytosis in neuroepithelial progenitor cells of zebrafish. Additionally, it proves that these techniques can be successfully applied to improve our knowledge of how endocytosis may affect the development of the CNS. For these reasons, the submitted paper is of primary interest for the readers of this journal.

Reviewer 3 Comments for the Author...

This paper provides a well-conceived and carefully conducted set of analyses of receptor-mediated endocytosis in neuroepithelial zebrafish cells with additional hints on Ocrl function in these progenitor cells. In my opinion, it fully deserves publication in this journal.
We thank the reviewer for their positive comments on our work.

Minor comments and suggested revisions:

(i) lines 178-179: "Collectively, these results demonstrate that RAP endocytosis at the neuroepithelium can be quantified and reliably detect the anticipated differences in endocytic uptake". The term "quantified" may be considered an overstatement since it can only be applied to Fig.2F, where differences between the rates of endocytosis occurring at various concentrations of the injected marker are statistically significant at the same time point. This observation cannot be extended to the other statistic analyses in Fig.2.

Henceforth, I would rephrase the sentences from line 170 to the end of the paragraph to reflect this consideration.
There are indeed no statistically significant differences between 2.5 and 1.25 mg/ml of injected RAP which we believe may be because concentrations of 1.25 mg/ml and above are saturating available RAP binding receptors for the time periods analysed. Statistically significant differences between 2.5-1.25 mg/ml and 0.625 mg/ml could be observed however, suggesting that larger differences in RAP uptake can be quantified and reach statistical significance. Based on the absence of significance between 2.5 mg/ml and 1.25 mg/ml concentrations of RAP however, we agree that the sensitivity of our method in picking up more subtle differences in RAP uptake may be limited. We have updated the text (page 7) to reflect these caveats which we hope satisfies the reviewer’s point.

© 2022. Published by The Company of Biologists under the terms of the Creative Commons Attribution License (https://creativecommons.org/licenses/by/4.0/).

(ii) lines 215-216: "In support of an effect on endosomal trafficking, EEA1-positive early endosomes were enlarged in ocrl mutant embryos...". The measurement of enlarged endosomes relies on confocal analyses. It would be more appropriate to use EM for this purpose. However, since these data are not crucial for the conclusions of this paper, I advise mentioning that ultrastructural analyses may be more appropriate to draw final conclusions on this topic.
We agree that EM analysis of endosome morphology and size within the *ocrl* neuroepithelium would likely provide a clearer result regarding the effect of loss of Ocrl function on neuroepithelial endosome size. We have updated the discussion (page 12) with reference to the use of ultrastructural analysis of endosomes by EM.

(iii) Fig. 4, panel A. The staining for Ocrla is very dim and raises doubts about a possible spillover/bleed-through of the Cy3 channel into the EGFP channel. I would suggest checking for EGFP-Ocrla expression, firstly (e.g., by WB). Then, if confirmed, you could either repeat the experiment and improve the EGFP-Ocrla detection or check whether there is any bleeding from the Cy3 channel into the EGFP channel by undertaking the appropriate controls.
We thank the reviewer for this observation. We are confident the labelled EGFP signal we show, although weaker than other markers used in this study, is from expression of EGFP- Ocrla in neuroepithelial cells (see Reviewer Fig 3A for background fluorescence control with no EGFP-Ocrla expression, compared to signal from expression of the EGFP-tagged protein). We also carefully controlled for bleed through from the Cy3 signal into the EGFP channel using tuneable mirrors on the confocal system. In Reviewer Figure 3B we have provided an example of bleed through of the Cy3 signal into the EGFP channel using sub- optimal settings which permit spill-over of signal from the brighter RAP-Cy3 puncta (494- 563nm, top panel) which may indeed be confused for signal from EGFP-Ocrla (orange arrowheads). Imaging the same region with a narrower range of collected emission wavelengths (494-545nm, bottom panel) limits bleed through from RAP-Cy3, with loss of multiple puncta from the EGFP channel. The remaining EGFP puncta (white arrowhead) are therefore specific to EGFP-Ocrla. These same settings were used to collect the representative images displayed in Fig. 4A of the manuscript.

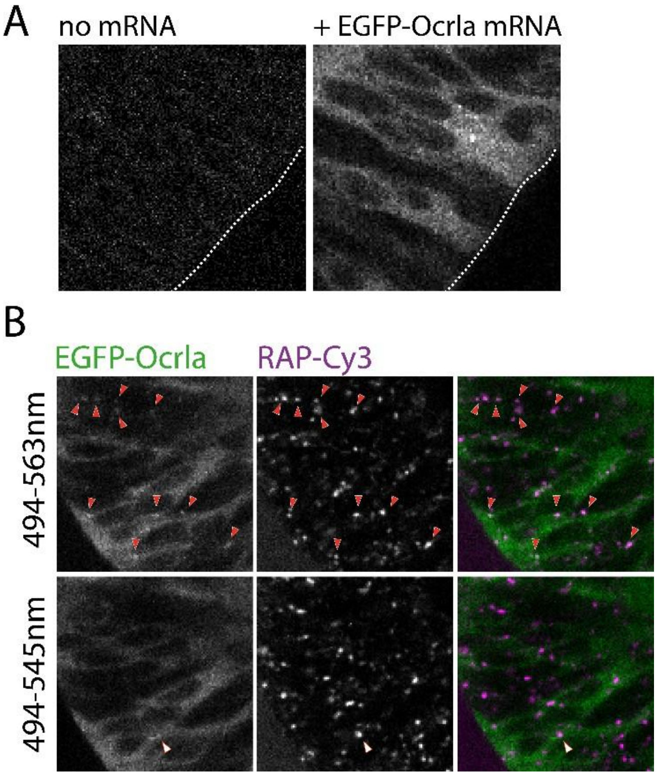

Reviewer Figure 3. A) Specificity control for EGFP-Ocrla signal in neuroepithelial cells. One cell stage embryos were left uninjected (left panel) or injected with mRNA encoding EGFP-Ocrla (right panel). Neuroepithelial cells from each group were imaged by confocal microscopy at 28hpf. **B)** Specificity control for EGFP-Ocrla signal in the presence of RAP-Cy3. A 28hpf embryo injected with

© 2022. Published by The Company of Biologists under the terms of the Creative Commons Attribution License (https://creativecommons.org/licenses/by/4.0/).

EGFP-Ocrla mRNA at the one cell stage was injected with RAP-Cy3 and imaged 1 hour post-injection. The same region of the MHB was imaged with emission wavelengths for EGFP collected between 494-563nm or 494-545nm.

References

Clark, B. S., M. Winter, A. R. Cohen and B. A. Link (2011). "Generation of Rab-based transgenic lines for in vivo studies of endosome biology in zebrafish." Dev Dyn **240**(11): 2452-2465.
Czekay, R. P., R. A. Orlando, L. Woodward, M. Lundstrom and M. G. Farquhar (1997). "Endocytic trafficking of megalin/RAP complexes: dissociation of the complexes in late endosomes." Mol Biol Cell **8**(3): 517-532.
Festa, B. P., M. Berquez, A. Gassama, I. Amrein, H. M. Ismail, M. Samardzija, L. Staiano, A. Luciani, C. Grimm, R. L. Nussbaum, M. A. De Matteis, O. M. Dorchies, L. Scapozza, D. P. Wolfer and O. Devuyst (2019). "OCRL deficiency impairs endolysosomal function in a humanized mouse model for Lowe syndrome and Dent disease." Hum Mol Genet **28**(12): 1931-1946.
Hsieh, J. C., L. Lee, L. Zhang, S. Wefer, K. Brown, C. DeRossi, M. E. Wines, T. Rosenquist and B. C. Holdener (2003). "Mesd encodes an LRP5/6 chaperone essential for specification of mouse embryonic polarity." Cell **112**(3): 355-367.
Lagache, T., Sauvonnet, N., Danglot, L., and Olivo-Marin, J.C. (2015). "Statistical analysis of molecule colocalization in bioimaging." Cytometry **87**, 568-579.
McCluskey, A., J. A. Daniel, G. Hadzic, N. Chau, E. L. Clayton, A. Mariana, A. Whiting, N. N. Gorgani, J. Lloyd, A. Quan, L. Moshkanbaryans, S. Krishnan, S. Perera, M. Chircop, L. von Kleist, A. B. McGeachie, M. T. Howes, R. G. Parton, M. Campbell, J. A. Sakoff, X. Wang, J. Y. Sun, M. J. Robertson, F. M. Deane, T. H. Nguyen, F. A. Meunier, M. A. Cousin and P. J. Robinson (2013). "Building a better dynasore: the dyngo compounds potently inhibit dynamin and endocytosis." Traffic **14**(12): 1272-1289.
Nagai, M., T. Meerloo, T. Takeda and M. G. Farquhar (2003). "The adaptor protein ARH escorts megalin to and through endosomes." Mol Biol Cell **14**(12): 4984-4996.
Oltrabella, F., A. Jackson-Crawford, G. Yan, S. Rixham, T. Starborg and M. Lowe (2021). "IPIP27A cooperates with OCRL to support endocytic traffic in the zebrafish pronephric tubule." Hum Mol Genet. Oltrabella, F., G. Pietka, I. B. Ramirez, A. Mironov, T. Starborg, I. A. Drummond, K. A. Hinchliffe and M. Lowe (2015). "The Lowe syndrome protein OCRL1 is required for endocytosis in the zebrafish pronephric tubule." PLoS Genet **11**(4): e1005058.
Perez Bay, A. E., R. Schreiner, I. Benedicto, M. Paz Marzolo, J. Banfelder, A. M. Weinstein and E. J. Rodriguez-Boulan (2016). "The fast-recycling receptor Megalin defines the apical recycling pathway of epithelial cells." Nat Commun 7: 11550.
Ramirez, I. B., Pietka, G., Jones, D. R., Divecha, N., Alia, A., Baraban, S. C., Hurlstone, A. F. and Lowe, M. (2012). "Impaired neural development in a zebrafish model for Lowe syndrome." Hum Mol Genet **21**, 1744-59.
Shah, M., O. Y. Baterina, Jr., V. Taupin and M. G. Farquhar (2013). "ARH directs megalin to the endocytic recycling compartment to regulate its proteolysis and gene expression." J Cell Biol **202**(1): 113-127.
Sonnichsen, B., S. De Renzis, E. Nielsen, J. Rietdorf and M. Zerial (2000). "Distinct membrane domains on endosomes in the recycling pathway visualized by multicolor imaging of Rab4, Rab5, and Rab11." J Cell Biol **149**(4): 901-914.
Vicinanza, M., A. Di Campli, E. Polishchuk, M. Santoro, G. Di Tullio, A. Godi, E. Levtchenko, M. G. De Leo, R. Polishchuk, L. Sandoval, M. P. Marzolo and M. A. De Matteis (2011). "OCRL controls trafficking through early endosomes via PtdIns4,5P(2)-dependent regulation of endosomal actin." EMBO J **30**(24): 4970-4985.

© 2022. Published by The Company of Biologists under the terms of the Creative Commons Attribution License (https://creativecommons.org/licenses/by/4.0/).

Second decision letter

MS ID#: JOCES/2022/260339

MS TITLE: Assessment of endocytic traffic and Ocrl function in the developing zebrafish neuroepithelium

AUTHORS: Daniel Williams, Lale Gungordu, Anthony Jackson-Crawford, and Martin Lowe
ARTICLE TYPE: Tools and Resources

I am happy to tell you that your manuscript has been accepted for publication in Journal of Cell Science, pending standard ethics checks.

© 2022. Published by The Company of Biologists under the terms of the Creative Commons Attribution License (https://creativecommons.org/licenses/by/4.0/).
